# SLC38A2 and glutamine signalling in cDC1s dictate anti-tumour immunity

**DOI:** 10.1038/s41586-023-06299-8

**Published:** 2023-07-05

**Authors:** Chuansheng Guo, Zhiyuan You, Hao Shi, Yu Sun, Xingrong Du, Gustavo Palacios, Cliff Guy, Sujing Yuan, Nicole M. Chapman, Seon Ah Lim, Xiang Sun, Jordy Saravia, Sherri Rankin, Yogesh Dhungana, Hongbo Chi

**Affiliations:** Department of Immunology, St. Jude Children’s Research Hospital, Memphis, TN USA

**Keywords:** Conventional dendritic cells, Immunosurveillance, Lymphocyte activation

## Abstract

Cancer cells evade T cell-mediated killing through tumour–immune interactions whose mechanisms are not well understood^1,2^. Dendritic cells (DCs), especially type-1 conventional DCs (cDC1s), mediate T cell priming and therapeutic efficacy against tumours^3^. DC functions are orchestrated by pattern recognition receptors^3–5^, although other signals involved remain incompletely defined. Nutrients are emerging mediators of adaptive immunity^6–8^, but whether nutrients affect DC function or communication between innate and adaptive immune cells is largely unresolved. Here we establish glutamine as an intercellular metabolic checkpoint that dictates tumour–cDC1 crosstalk and licenses cDC1 function in activating cytotoxic T cells. Intratumoral glutamine supplementation inhibits tumour growth by augmenting cDC1-mediated CD8^+^ T cell immunity, and overcomes therapeutic resistance to checkpoint blockade and T cell-mediated immunotherapies. Mechanistically, tumour cells and cDC1s compete for glutamine uptake via the transporter SLC38A2 to tune anti-tumour immunity. Nutrient screening and integrative analyses show that glutamine is the dominant amino acid in promoting cDC1 function. Further, glutamine signalling via FLCN impinges on TFEB function. Loss of FLCN in DCs selectively impairs cDC1 function in vivo in a TFEB-dependent manner and phenocopies SLC38A2 deficiency by eliminating the anti-tumour therapeutic effect of glutamine supplementation. Our findings establish glutamine-mediated intercellular metabolic crosstalk between tumour cells and cDC1s that underpins tumour immune evasion, and reveal glutamine acquisition and signalling in cDC1s as limiting events for DC activation and putative targets for cancer treatment.

## Abstract (web summary)

Competition for glutamine between type-1 conventional dendritic cells and tumour cells has a central role in tuning the anti-tumour immune response and in immune evasion by cancer cells.

## Main

Nutrients shape immune cell function and subset differentiation^6–8^, but our understanding of nutrient-mediated immune responses in different tissue microenvironments is limited. Nutrient and metabolite alterations occur in the tumour microenvironment (TME) and affect tumour–immune interactions^9^. For instance, altered glucose or amino acid composition contributes to dysregulated T cell effector function^10–14^ or myeloid cell activity^7^. In the TME, DCs capture and present tumour-associated antigens and provide costimulatory signals and soluble factors to provoke anti-tumour immunity^3^. DCs are activated upon sensing of environmental cues, including signals from dying tumour cells^3^. However, despite the emerging roles for intracellular metabolic pathways in mediating quiescence and activation of DCs^15–17^, whether and how nutrients or metabolic processes modulate DC function and heterogeneity, especially in the TME, remain largely unknown.

cDCs comprise cDC1 and cDC2 subsets, which promote CD8^+^ cytotoxic T cell and CD4^+^ T cell priming, respectively^4,5^. cDC1s also cross-present tumour-associated antigens to cytotoxic CD8^+^ T cells, thereby promoting anti-tumour immunity. Moreover, cDC1s aid in the success of immune checkpoint blockade (ICB) and adoptive T cell therapies^3,18–20^ (ACT). However, the immunosuppressive TME contributes to immune evasion via impairment of DC functional activity, partly mediated by dysregulated abundance or sensing of local environmental cues^3^. Pattern recognition receptors and other receptor systems sense environmental signals for functional specialization of DC subsets^3–5^, yet roles for nutrients and metabolites in this process are largely unresolved, especially compared with those in lymphocytes^6^. Uncovering mechanisms that mediate DC function or dysfunction in the TME will be crucial for effective cancer immunotherapy.

## Glutamine primes tumour immunity of cDC1s

**Fig. 1:**
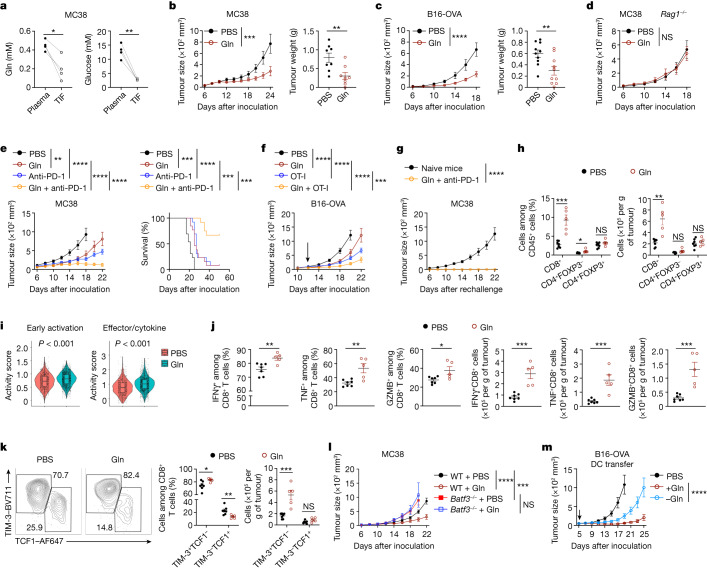
Intratumoral glutamine supplementation promotes cDC1-mediated anti-tumour immunity. **a**, Levels of glutamine and glucose in plasma and TIF of mice bearing MC38 tumours at day 15 (*n* = 4 per group). **b**,**c**, Growth and endpoint weight of MC38 (**b**; *n* = 8 per group) and B16-OVA (**c**; *n* = 10 per group) tumours (day 24 and 18, respectively) after intratumoral PBS or glutamine supplementation. **d**, MC38 tumour growth in *Rag1*^−/−^ mice after PBS or glutamine treatment (*n* = 7 per group). **e**, MC38 tumour growth and mouse survival after indicated treatments (*n* = 12 for Gln + anti-PD-1, *n* = 13 for all other groups). **f**, Growth of B16-OVA tumours in mice receiving intratumoral PBS or glutamine with activated OT-I cells (indicated by arrow) (*n* = 10 per group). **g**, MC38 tumour growth in tumour-free (having received prior glutamine + anti-PD-1 treatment; *n* = 8) or naive mice (*n* = 5) upon challenge with MC38 cells. **h**, Indicated T cell populations at day 15 in MC38 tumours treated with PBS (*n* = 7) or glutamine (*n* = 5). **i**, DCs, CD45^+^ non-macrophage immune cells, macrophages and CD45^−^ cells were sorted from PBS- and glutamine-treated MC38 tumours and mixed for scRNA-seq analysis. Violin plots show activity scores of early activation and effector/cytokine signalling signatures in intratumoral CD8^+^ T cells from MC38 tumours treated with PBS (*n* = 1,113 cells) or glutamine (*n* = 2,031 cells). Box plots show the median (centre line) with interquartile range of 25% to 75%. **j**,**k**, IFNγ^+^, TNF^+^ and granzyme B^+^ (GZMB^+^) (**j**) or effector-like (TIM-3^+^TCF1^−^) and stem-like (TIM-3^−^TCF1^+^) (**k**) CD8^+^ T cells at day 15 from MC38 tumours treated with PBS (*n* = 7) or glutamine (*n* = 5). **l**, MC38 tumour growth in indicated mice treated with PBS (*n* = 10 for wild-type, *n* = 8 for *Batf3*^−/−^) or glutamine (*n* = 9 for wild-type, *n* = 8 for *Batf3*^−/−^). WT, wild-type. **m**, Growth rate of B16-OVA tumours after transfer of OVA-pulsed cDC1s activated in the presence or absence of glutamine (*n* = 9 for DCs treated with glutamine, *n* = 8 for DCs treated without glutamine). Non-transfer control mice (*n* = 10) received PBS. Data are mean ± s.e.m., except in **i**. **a**, Two-tailed paired Student’s *t*-test. **b**,**c**,**h**,**j**,**k**, Two-tailed unpaired Student’s *t*-test (**b**,**c**, tumour weight). **b**–**g**,**l**,**m**, Two-way ANOVA for tumour size. **e**, Mantel–Cox test for survival. **i**, Two-tailed Wilcoxon rank sum test. Data are representative of two (**a**,**d**–**h**,**j**,**l**,**m**) or at least three (**b**,**c**,**k**) independent experiments. *P < 0.05, **P < 0.01, ***P < 0.001, ****P < 0.0001. NS, not significant. Source data

**Extended Data Fig. 1:**
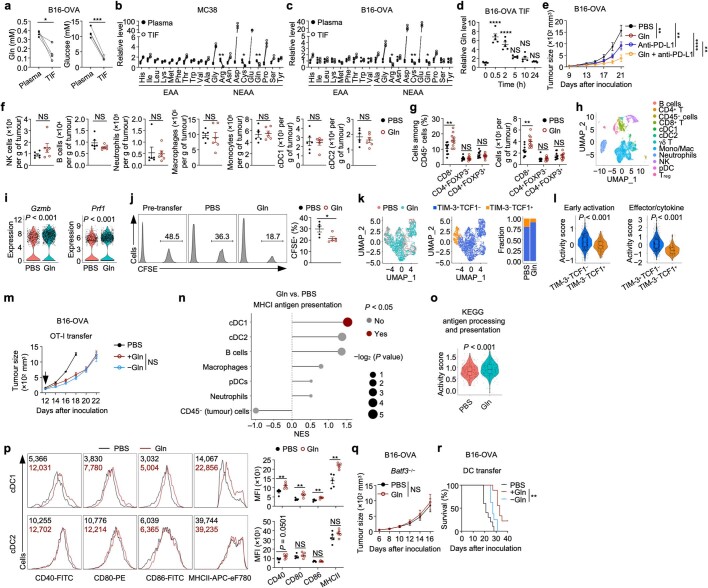
Glutamine supplementation enhances anti-tumour immunity. **a**, Glutamine (Gln) and glucose levels in plasma and tumour interstitial fluid (TIF) of B16-OVA tumour-bearing mice at day 15 (*n* = 4 each group). **b**,**c**, Amino acid levels in plasma and TIF of MC38 (**b**) or B16-OVA (**c**) tumour-bearing mice at day 15 (*n* = 4 each group). Cys, cysteine or cystine. **d**, Relative glutamine levels across time in B16-OVA TIF after intratumoral glutamine supplementation (*n* = 5 each group). **e**, B16-OVA tumour growth in mice receiving indicated treatments (*n* = 9 for Gln treatment; 10 for other groups). **f**, Indicated cell populations in PBS- or Gln-treated MC38 tumours at day 15 (*n* = 6 each group). **g**, Indicated T cell populations in PBS- or Gln-treated B16-OVA tumours at day 15 (*n* = 10 each group). **h**,**i**, DCs, CD45^+^ non-macrophage immune cells, macrophages, and CD45^−^ cells were sorted from PBS- and Gln-treated MC38 tumours and mixed at 5:4:1:1 ratio for scRNA-seq analysis. UMAP plot of cell subclusters (**h**). Violin plots showing *Gzmb* and *Prf1* expression in intratumoral CD8^+^ T cells (**i**). **j**, In vivo killing of OVA_257–264_-pulsed splenocytes (CFSE^+^) in MC38-OVA tumour-bearing mice treated with PBS or Gln (*n* = 5 each group). **k**, UMAP plots showing distribution of intratumoral CD8^+^ T cells subclusters in PBS- and Gln-treated MC38 tumours. Right, TIM-3^−^TCF1^+^ and TIM-3^+^TCF1^−^ CD8^+^ T cell subcluster quantification. **l**, Violin plots showing activity scores of early activation and effector/cytokine signalling gene signatures in TIM-3^+^TCF1^−^ and TIM-3^−^TCF1^+^ CD8^+^ T cells. **m**, B16-OVA tumour growth after receiving activated OT-I cells expanded in medium with or without Gln (*n* = 6 each group; adoptive transfer indicated by an arrow). Non-transfer control mice (*n* = 4) received PBS. **n**, The enrichment of the MHCI antigen presentation gene set in indicated cell populations (from scRNA-seq in **h**) as assessed by GSEA. NES, normalized enrichment score. **o**, Violin plot showing activity score of indicated gene signature in intratumoral cDC1 from PBS- or Gln-treated MC38 tumours. **p**, CD40, CD80, CD86, and MHCII expression on intratumoral cDC1s and cDC2s from PBS- or Gln-treated MC38 tumours at day 15 (*n* = 5 each group). MFI, mean fluorescence intensity. **q**, B16-OVA tumour growth in *Batf3*^−/−^ mice treated with intratumoral PBS or Gln (*n* = 8 per group). **r**, B16-OVA tumour-bearing mouse survival after transfer of OVA-pulsed cDC1 activated in presence or absence of Gln (*n* = 9 for DC treated with Gln; 8 for DC treated without Gln). Non-transfer control mice (*n* = 10) received PBS. Data are means ± s.e.m. except in **i**, **l**, **o**, where box shows median (centre line) with interquartile range of 25% to 75%. **a**–**c**, Two-tailed paired Student’s *t*-test. **f**,**g**,**j**,**p**, Two-tailed unpaired Student’s *t*-test. **d**, One-way ANOVA. **e**,**m**,**q**, Two-way ANOVA. **i**,**l**,**o**, Two-tailed Wilcoxon rank sum test. **n**, Two-tailed Kolmogorov–Smirnov test. **r**, Mantel**–**Cox test. Data are representative of one (**d**,**j**) or two (**a**–**c**, **e**–**g**, **m**, **p**–**r**) independent experiments. *P < 0.05, **P < 0.01, ***P < 0.001, **** *P* < 0.0001. NS, not significant. Source data

To examine the nutrient composition in the TME, we isolated tumour interstitial fluid (TIF) and matched plasma from mice challenged with MC38 colon adenocarcinoma cells or ovalbumin (OVA)-expressing B16F10 (B16-OVA) melanoma cells and performed metabolomics profiling. Glucose, glutamine, arginine and cysteine (or cystine) were reduced in TIF compared with matched plasma (Fig. 1a and Extended Data Fig. 1a–c). The decrease in glutamine was of particular interest, as pharmacological blockade of glutamine metabolism in tumour-bearing mice or glutaminase deletion in tumour cells suppresses cancer cell growth and enhances CD8^+^ T cell anti-tumour activity^21,22^. However, glutaminase inhibition also impairs CD8^+^ T cell activation and anti-tumour capabilities in the TME^23^. On the basis of the observations above, we tested whether directly enhancing intratumoral glutamine abundance affects anti-tumour immunity. Daily intratumoral glutamine injections of B16-OVA tumour-bearing mice increased glutamine levels in TIF (Extended Data Fig. 1d). Notably, such treatments reduced tumour growth and weight in wild-type mice challenged with MC38 or B16-OVA tumour cells but not in *Rag1*^–/–^ mice (Fig. 1b–d), suggesting the importance of adaptive immunity in mediating the anti-tumour effect of glutamine.

As resistance to immunotherapy remains a key challenge for cancer treatment^1,2^, we next examined whether intratumoral glutamine supplementation improves ICB responses. Combinatorial treatment with glutamine enhanced the efficacy of anti-PD-1-mediated ICB therapy, with the majority (67%) of combination therapy-treated mice completely rejecting MC38 tumours and surviving (Fig. 1e). Similar effects were observed on B16-OVA tumour growth (Extended Data Fig. 1e). Glutamine supplementation also boosted ACT efficacy mediated by OVA-specific CD8^+^ T (OT-I) cells (Fig. 1f). Finally, on day 60 after tumour clearance, we rechallenged tumour-free mice that were previously treated with glutamine plus anti-PD-1 with MC38 cells, and found that tumours were completely rejected (Fig. 1g), indicating induction of strong immunological memory. Thus, intratumoral glutamine supplementation represents a powerful means to impede tumour growth and bolster cancer immunotherapy.

Next, we determined whether glutamine supplementation affects adaptive immune profiles. In mice supplemented with glutamine, the frequency and number of intratumoral CD8^+^ T cells were increased, with modest or no effect on other lymphocyte and myeloid populations (Fig. 1h and Extended Data Fig. 1f,g). We then performed single-cell RNA sequencing (scRNA-seq) to profile intratumoral CD45^+^ immune cells—which included CD8^+^ T cells, FOXP3^–^CD4^+^ T cells, FOXP3^+^CD4^+^ T regulatory (T_reg_) cells, cDC1s, cDC2s and plasmacytoid DCs (pDCs) (Extended Data Fig. 1h)—and CD45^–^ non-immune (predominantly tumour) cells from PBS or glutamine-treated MC38 tumours for mechanistic insights (Methods). Intratumoral CD8^+^ T cells from glutamine-treated mice showed increased activity scores for gene signatures related to CD8^+^ T cell early activation and effector/cytokine signalling (Fig. 1i), as well as augmented expression of *Gzmb* (which encodes granzyme B) and *Prf1* (encoding perforin) (Extended Data Fig. 1i), indicative of improved effector function. Accordingly, glutamine-supplemented mice had increased percentages and numbers of intratumoral CD8^+^ T cells that expressed IFNγ, TNF or granzyme B (Fig. 1j), and more readily cleared tumour antigen-pulsed splenocytes in an in vivo killing assay^24^ (Extended Data Fig. 1j). Recent studies unveil the heterogeneity and dynamics of CD8^+^ T cells in cancer and chronic viral infections, including TCF1^–^ effector-like cells that upregulate expression of TIM-3^25,26^ (encoded by *Havcr2*). scRNA-seq analysis revealed that intratumoral CD8^+^ T cells from glutamine-supplemented mice had a greater proportion of TIM-3^+^TCF1^–^ effector-like cells (Extended Data Fig. 1k), characterized by the expected activation state and effector function^27,28^ (Extended Data Fig. 1l), and flow cytometry analysis further validated these results (Fig. 1k). Therefore, glutamine supplementation boosts the accumulation and effector function of intratumoral CD8^+^ T cells.

To determine whether glutamine supplementation exerts T cell-intrinsic anti-tumour immunity, we generated activated OT-I cells that were expanded in medium lacking glutamine, followed by adoptive transfer into B16-OVA tumour-bearing mice. These cells retained the ability to control tumour growth (Extended Data Fig. 1m), suggesting that glutamine is likely to influence intratumoral T cell function via cell-extrinsic effects. Myeloid cells control anti-tumour immunity by presenting tumour-associated antigens to T cells^3^. Among the cells that may serve as antigen-presenting cells for intratumoral T cells (Extended Data Fig. 1h), cDC1s from glutamine-treated mice showed significantly increased activity scores of major histocompatibility complex class I (MHCI) antigen presentation signature and antigen processing and presentation pathway, as revealed by gene set enrichment analysis (GSEA) of our scRNA-seq dataset  (Extended Data Fig. 1n,o). Moreover, intratumoral cDC1s but not cDC2s had increased expression of CD40, CD80, CD86 and MHCII in glutamine-supplemented mice (Extended Data Fig. 1p). Thus, glutamine supplementation enhances the maturational state of intratumoral cDC1s.

Notably, MC38 and B16-OVA tumour growth was undisturbed by glutamine supplementation in *Batf3*^*–/–*^ mice that lack cDC1s^29^ (Fig. 1l and Extended Data Fig. 1q), indicating that the beneficial effect of glutamine treatment requires cDC1s. To test whether glutamine affects cDC1-mediated vaccine efficacy, we adopted a DC transfer model for tumour therapy^30^ by pulsing splenic cDC1s with OVA and poly I:C in glutamine-sufficient or -deficient medium prior to injection into B16-OVA tumour-bearing mice. cDC1s pulsed in glutamine-deficient medium exhibited an impaired therapeutic effect (Fig. 1m and Extended Data Fig. 1r). Thus, glutamine is essential and limiting for enabling the anti-tumour activity of both endogenous and adoptively transferred cDC1s, suggesting an interplay between glutamine and cDC1s for anti-tumour immunity.

**Extended Data Fig. 2:**
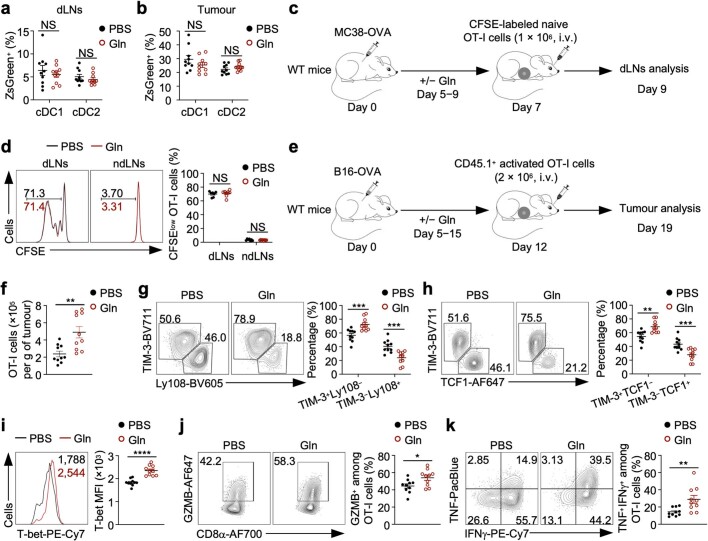
Glutamine supplementation promotes intratumoral CD8^+^ T cell activation and effector function. **a**,**b**, ZsGreen^+^ migratory cDC1s and cDC2s in tumour-draining lymph nodes (dLNs; **a**) or tumour (**b**) of PBS- or Gln-treated B16-ZsGreen tumour-bearing mice at day 16 (*n* = 10 each group). Gln, glutamine. **c**,**d**, Schematic depicting adoptive transfer of CFSE-labelled naïve OT-I cells into MC38-OVA tumour-bearing mice treated with PBS (*n* = 7) or Gln (*n* = 8) (**c**). CFSE^low^ OT-I cells in tumour dLNs and non-dLNs (ndLNs) (**d**). **e**–**k** Schematic depicting adoptive transfer of CD45.1^+^ activated OT-I cells into B16-OVA tumour-bearing mice treated with PBS or Gln (*n* = 10 each group) (**e**). Intratumoral OT-I cell number (**f**). Effector-like (TIM-3^+^Ly108^−^ or TIM-3^+^TCF1^−^) and stem-like (TIM-3^−^Ly108^+^ or TIM-3^−^TCF1^+^) (**g**,**h**) subsets of OT-I cells. T-bet mean fluorescence intensity (MFI) in OT-I cells (**i**). Granzyme B^+^ (GZMB^+^) (**j**) and TNF^+^IFNγ^+^ (**k**) OT-I cells. Data are means ± s.e.m. **a**,**b**,**d**,**f**,**i**–**k**, Two-tailed unpaired Student’s *t*-test. **g**,**h**, Two-way ANOVA. Data are from one experiment (**a**,**b**,**d**,**f**–**k**). *P < 0.05, **P < 0.01, ***P < 0.001, ****P < 0.0001. NS, not significant. Source data

To explore the potential mechanisms, we first assessed whether intratumoral glutamine supplementation affects DC migration to tumour-draining lymph nodes (dLNs), by using B16F10 tumour cells expressing the fluorescent protein ZsGreen (B16-ZsGreen)^31^. The percentages of ZsGreen^+^ migratory DCs in tumour dLNs and ZsGreen^+^ DCs in tumours were unaltered by glutamine supplementation (Extended Data Fig. 2a,b), indicating the lack of effect on DC tumour antigen uptake or migratory capacity. To determine whether cross-priming is altered in tumour dLNs, MC38-OVA tumour-bearing mice treated with intratumoral glutamine were given adoptive transfer of carboxyfluoroscein succinimidyl ester (CFSE)-labelled naive OT-I cells^31^, and assessment of proliferation revealed no impairment (Extended Data Fig. 2c, d). Thus, glutamine supplementation had no effect on naive CD8^+^ T cell priming in tumour dLNs. To assess the capacity of effector CD8^+^ T cells to respond to antigen restimulation in the TME, we adoptively transferred activated OT-I cells into B16-OVA tumour-bearing mice and assessed their activation in the TME (Extended Data Fig. 2e). OT-I cells with effector-like phenotypes showed greater accumulation in glutamine-treated tumours (Extended Data Fig. 2f–h), and also showed increased T-bet expression and higher frequencies of granzyme B- or IFNγ- and TNF-co-expressing cells (Extended Data Fig. 2i–k). Collectively, intratumoral glutamine supplementation enhances CD8^+^ T cell effector function in the TME without affecting DC migration or naive CD8^+^ T cell priming.

## SLC38A2 dictates tumour–cDC1 crosstalk

**Fig. 2:**
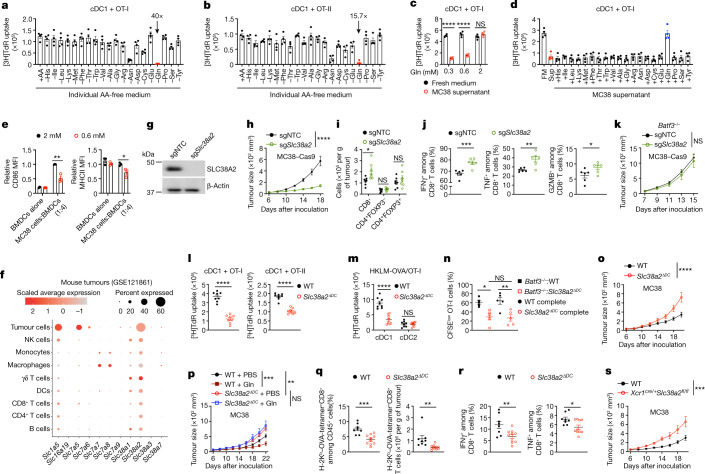
Glutamine interplay between tumour cells and cDC1s modulates anti-tumour immunity. **a**–**d**, [^3^H]Thymidine (TdR) incorporation by OT-I (**a**,**c**,**d**) or OT-II (**b**) cells after coculture with cDC1s (*n* = 4 per group) pulsed with OVA in amino acid (AA)-replete medium (+AA) or medium lacking an individual amino acid (**a**,**b**), in culture supernatants derived from MC38 cells cultured in glutamine-free medium supplemented with indicated glutamine concentrations (**c**), or in MC38 culture supernatant supplemented with an individual amino acid (**d**). Arrows in **a**,**b** indicate fold change for +AA versus −Gln. **e**, CD86 and MHCII expression on BMDCs after 24 h of Transwell coculture with MC38 cells in medium containing 2 or 0.6 mM glutamine (*n* = 3 per group). MFI, mean fluorescence intensity. **f**, Expression of glutamine transporters in indicated mouse cell types (from GSE121861). NK cells, natural killer cells. **g**, Immunoblot analysis of SLC38A2 and β-actin in control and SLC38A2-deficient MC38 cells. **h**, Growth of control and SLC38A2-deficient MC38 tumours in wild-type mice (*n* = 10 per group). **i**,**j**, Indicated T cell populations (**i**) or IFNγ^+^, TNF^+^ or granzyme B^+^ (GZMB^+^) CD8^+^ T cells (**j**) from control and SLC38A2-deficient MC38 tumours at day 15 (*n* = 7 (**i**) or 6 (**j**) per group). **k**, Growth of control and SLC38A2-deficient MC38 tumours in *Batf3*^−/−^ mice (*n* = 10 per group). **l**,**m**, [^3^H]TdR incorporation by OT-I (**l**,**m**) or OT-II (**l**) cells after coculture with OVA-pulsed (**l**) or HKLM-OVA-pulsed (**m**) wild-type and SLC38A2-deficient cDC1s (*n* = 8 per genotype). **n**, CFSE^low^ OT-I cells in indicated OVA-immunized chimeric mice at day 3 after challenge (*n* = 5 per group). **o**,**p**, Growth of MC38 tumours in wild-type (*n* = 10) and *Slc38a2*^*ΔDC*^ (*n* = 9) mice (**o**) or in mice given intratumoral PBS (*n* = 9 for wild-type mice, *n* = 8 for *Slc38a2*^*ΔDC*^ mice) or glutamine (*n* = 7 per genotype) (**p**). **q**,**r**, OVA-specific CD8^+^ T cells (**q**) or IFNγ^+^ and TNF^+^ CD8^+^ T cells (**r**) in MC38-OVA tumours from wild-type (*n* = 8) and *Slc38a2*^*ΔDC*^ (*n* = 10) mice at day 19. **s**, MC38 tumour growth in wild-type (*n* = 7) and *Xcr1*^*cre/+*^*Slc38a2*^*fl/fl*^ (*n* = 5) mice. Data are mean ± s.e.m. **c**,**e**,**i**,**j**,**l**,**m**,**q**,**r**, Two-tailed unpaired Student’s *t*-test. **n**, One-way ANOVA. **h**,**k**,**o**,**p**,**s**, Two-way ANOVA. Data are representative of one (**g**), two (**e**,**i**–**k**,**m**–**s**) or at least three (**a**–**d**,**h**,**l**) independent experiments. *P < 0.05, **P < 0.01. ***P < 0.001, ****P < 0.0001. NS, not significant. Source data

**Extended Data Fig. 3:**
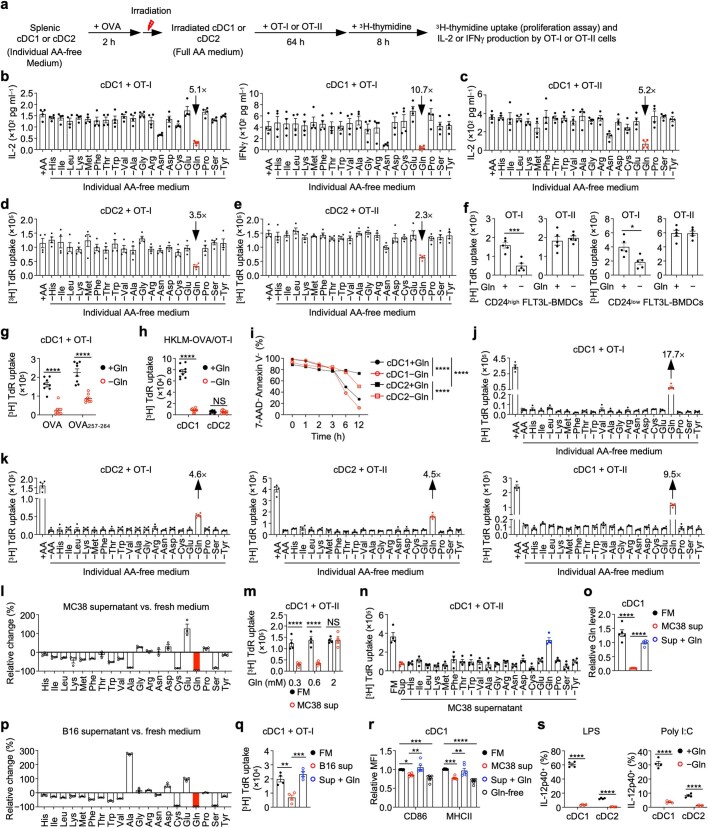
Glutamine promotes priming capacity of DCs. **a**, Schematic of functional amino acid screening assay used in Fig. 2a, b and Extended Data Fig. 3b–e. **b**–**e**, IL-2 (**b**,**c**) and IFNγ (**b**) production or [^3^H] Thymidine (TdR) incorporation by OT-I (**b**,**d**) or OT-II (**c**,**e**) cells after coculture with splenic cDC1s (**b**,**c**) or cDC2s (**d**,**e**) pulsed with OVA in amino acid-replete medium (+AA) or medium lacking an individual amino acid (*n* = 4 each group). Arrow, fold change (+AA versus −Gln) (*n* = 4 each group). Gln, glutamine. **f**, [^3^H] TdR incorporation by OT-I or OT-II cells after coculture with cDC1 (CD24^high^)- and cDC2 (CD24^low^)-like FLT3L-BMDCs pulsed with OVA in medium with or without Gln (*n* = 5 each group). BMDCs, bone marrow-derived DCs. **g**,**h**, [^3^H] TdR incorporation by OT-I cells after coculture with splenic cDC1s pulsed with OVA or OVA_257–264_ (**g**) or HKLM-OVA (**h**) in Gln-replete or -deficient medium (**g**, *n* = 8 each group; **h**, *n* = 9 each group). **i**, Live (7-AAD^−^Annexin V^−^) splenic cDC1s and cDC2s after culture with or without Gln for indicated times (*n* = 4 per group). **j**,**k**, [^3^H] TdR incorporation by OT-I or OT-II cells after coculture with cDC1s (**j**) or cDC2s (**k**) pulsed with OVA in Gln-free medium (–AA) supplemented with an individual amino acid (*n* = 4 each group). Arrow, fold change (+Gln versus −AA). **l**, Relative changes in amino acid levels in MC38 cell culture supernatant versus fresh medium (FM; containing 0.6 mM Gln) (*n* = 3 per group). Red bar indicates Gln. **m**,**n**, [^3^H] TdR incorporation by OT-II cells after coculture with cDC1s pulsed with OVA in FM or the medium supernatant (MC38 sup) derived from MC38 cells cultured in glutamine-free medium supplemented with indicated glutamine concentrations (**m**) or supernatant supplemented with an individual amino acid (**n**) (*n* = 4 each group). **o**, Intracellular Gln levels in splenic cDC1s cultured under indicated conditions (*n* = 5 each group). **p**, Relative changes in amino acid levels in B16F10 cell culture supernatant versus fresh medium (containing 0.6 mM Gln) (*n* = 3 each group). Red bar indicates Gln. **q**, [^3^H] TdR incorporation by OT-I cells after coculture with splenic cDC1s pulsed with OVA in FM or B16F10 cell culture supernatant (B16 sup) supplemented with or without Gln (*n* = 4 each group). **r**, CD86 and MHCII expression on cDC1s incubated in the indicated media (*n* = 6 each group). MFI, mean fluorescence intensity. **s**, IL-12p40^+^ cDC1s and cDC2s after lipopolysaccharide (LPS) or poly I:C stimulation in Gln-replete or -deficient medium (*n* = 4 each group). Data are means ± s.e.m. **f**–**h**,**m**,**s**, Two-tailed unpaired Student’s *t*-test. **o**,**q**,**r**, One-way ANOVA. Data are representative of one (**l**,**p**), two (**f**–**i**,**q**) or at least three (**b**–**e**,**j**,**k**,**m**–**o**,**s**), or pooled from three (**r**) independent experiments. *P < 0.05, **P < 0.01, ***P < 0.001, ****P < 0.0001. NS, not significant. Source data

Next, we performed a comprehensive nutrient screening assay to determine how glutamine affects DC function compared with other amino acids, using priming of OT-I or OT-II (OVA-specific CD4^+^) cells as DC functional readouts^16^ (Extended Data Fig. 3a). Glutamine deprivation of cDC1s, and to a lesser extent cDC2s, reduced OT-I or OT-II cell proliferation and IL-2 or IFNγ production (Fig. 2a,b and Extended Data Fig. 3b–e). Similarly, CD8^+^ T cell priming by bone marrow-derived DCs (BMDCs) cultured in the FLT3L system was impaired upon glutamine depletion (Extended Data Fig. 3f). Glutamine-deprived cDC1s were also defective in promoting OT-I cell proliferation in response to OVA_257–264_, albeit to a lesser extent than in response to OVA (Extended Data Fig. 3g), suggesting a stronger impairment in antigen cross-presentation. Further, glutamine-deprived cDC1s showed reduced cross-presentation of heat-killed OVA-expressing *Listeria monocytogenes* (HKLM-OVA) to OT-I cells (Extended Data Fig. 3h), thereby indicating an essential role for glutamine in cDC1-mediated priming of CD8^+^ T cells. Notably, glutamine starvation for up to 3 h—the time frame used for all in vitro assays—had minimal effect on the survival of cDC1s and cDC2s, although longer durations caused more death (Extended Data Fig. 3i), indicating that defective T cell priming by glutamine-deprived DCs was not attributed to increased cell death. Finally, we pulsed cDC1s or cDC2s with OVA in amino acid-free medium supplemented with an individual amino acid, and then cocultured them with OT-I or OT-II cells. Supplementation with glutamine alone enabled cDC1 (and to a lesser extent cDC2)-dependent T cell proliferation (Extended Data Fig. 3j,k). Thus, glutamine is both necessary and sufficient for supporting DC function in mediating T cell priming, with a preferential effect observed in cDC1s compared with cDC2s.

Given these results, we tested whether nutrient communication between tumour cells and DCs restricts DC access to glutamine and functional capacity. MC38 cells cultured with a physiological concentration (0.6 mM) of glutamine^32^ exhibited reduced glutamine levels in the culture supernatant (Extended Data Fig. 3l). cDC1s pulsed with OVA in such supernatant had an impaired capacity to prime T cell proliferation, and supplementation of glutamine, but not other amino acids, to MC38 supernatant rectified the priming defects (Fig. 2c,d and Extended Data Fig. 3m,n). Accordingly, glutamine supplementation restored intracellular glutamine abundance in cDC1s treated with MC38 supernatant (Extended Data Fig. 3o). Similar results were obtained using glutamine-supplemented B16F10 culture supernatant (Extended Data Fig. 3p,q). Glutamine deprivation by tumour cells probably impairs DC functional activation, as cDC1s cultured in MC38 supernatant showed reduced CD86 and MHCII expression, which was restored by glutamine supplementation, and glutamine-deprived DCs also showed decreased IL-12p40 production upon stimulation of pattern recognition receptors (Extended Data Fig. 3r,s). To directly test whether tumour cells outcompete DCs for glutamine, we cocultured in vitro-derived immature DCs with MC38 cells in a Transwell, thereby allowing only soluble factors to affect DC maturation (Methods). In medium containing 2 mM glutamine, addition of MC38 cells induced upregulation of CD86, which was impaired in the presence of a physiological concentration (0.6 mM) of glutamine. We also observed impaired MHCII expression (Fig. 2e). Therefore, tumour cells appear to outcompete DCs for glutamine, impairing their maturation and function.

**Extended Data Fig. 4:**
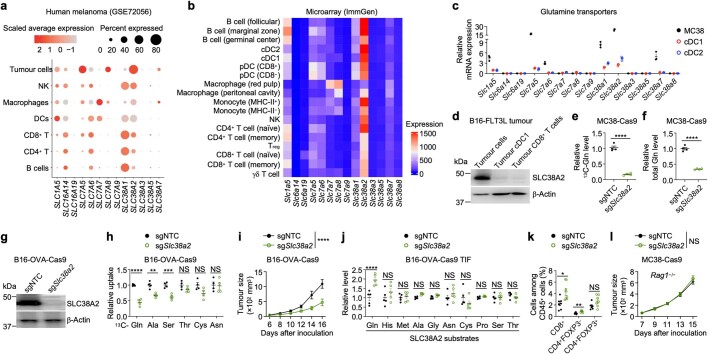
SLC38A2 deficiency in tumour cells promotes anti-tumour immunity. **a**, Expression of glutamine transporters in indicated human cell populations (from GSE72056). **b**, Microarray analysis (from ImmGen database) showing the relative expression of the indicated glutamine transporters across different immune cell types. **c**, Real-time PCR analysis of mRNA levels of glutamine transporters in MC38 cells, cDC1s and cDC2s (*n* = 3 each group). **d**, Immunoblot analysis of SLC38A2 and β-Actin in intratumoral cDC1s, CD8^+^ T and tumour cells from B16-FLT3L tumours. kDa, kilodaltons. **e**, Intracellular ^13^C-glutamine (Gln) levels in control and SLC38A2-deficient MC38 cells after 10 min incubation (*n* = 4 each group). **f**, Intracellular Gln levels in control and SLC38A2-deficient MC38 cells (*n* = 4 each group). **g**, Immunoblot analysis of SLC38A2 and β-Actin in control and SLC38A2-deficient B16-OVA cells. **h**, Intracellular levels of indicated ^13^C-labelled amino acids control and SLC38A2-deficient B16-OVA cells after 10 min incubation (*n* = 4 for each genotype). Ala, alanine; Ser, serine; Thr, threonine; Cys, cysteine; Asn, asparagine. **i**, Growth of control (*n* = 7) or SLC38A2-deficient (*n* = 9) B16-OVA tumours in wild-type mice. **j**, Levels of indicated SLC38A2 substrates in tumour interstitial fluid (TIF) from control and SLC38A2-deficient B16-OVA tumours at day 15 (*n* = 5 each group). Cys, cysteine or cystine. **k**, Indicated T cell populations in control and SLC38A2-deficient MC38 tumours at day 15 (*n* = 7 each group). **l**, Control and SLC38A2-defcient tumour growth in *Rag1*^−/−^ mice (*n* = 14 each group). Data are means ± s.e.m. **e**,**f**,**h**,**k**, Two-tailed unpaired Student’s *t*-test. **i**,**j**,**l**, Two-way ANOVA. Data are representative of one (**c**,**d**,**g**,**j**) or two (**e**,**f**,**h**,**i**,**k**, **l**) independent experiments. *P < 0.05, **P < 0.01, ****P < 0.0001. NS, not significant. Source data

To establish the underlying mechanisms, we analysed the expression of solute carrier (SLC) family members implicated in glutamine transport^33^ (Supplementary Table 1) in tumour cells and various immune cells, using publicly available syngeneic mouse tumour^34^ and human melanoma^35^ datasets. *SLC38A2* transcripts were highly expressed in both mouse and human tumour cells (Fig. 2f and Extended Data Fig. 4a). In the ImmGen database, *Slc38a2* was broadly expressed in multiple immune cells, with DCs expressing higher levels of *Slc38a2* than T cells and macrophages (Extended Data Fig. 4b). Tumour cells expressed higher *Slc38a2* transcript levels than DCs, CD8^+^ T or other immune cells (Fig. 2f and Extended Data Fig. 4a). Real-time PCR analysis validated abundant *Slc38a2* expression, with higher levels observed in MC38 cells compared with cDC1s or cDC2s (Extended Data Fig. 4c). Further, tumour cells expressed higher SLC38A2 protein levels than intratumoral cDC1s and CD8^+^ T cells (Extended Data Fig. 4d). Thus, SLC38A2 represents a putative intercellular metabolic checkpoint for dictating glutamine uptake and downstream functions between tumour cells and cDC1s.

To test this hypothesis, we generated SLC38A2-deficient tumour cells using single guide RNA (sgRNA) targeting *Slc38a2* (sg*Slc38a2*)—or non-targeting control (sgNTC)—to delete SLC38A2 in Cas9-expressing MC38 cells (Fig. 2g). In SLC38A2-deficient MC38 cells, [^13^C]glutamine uptake and total intracellular glutamine levels were reduced (Extended Data Fig. 4e,f), indicative of impaired glutamine uptake. Further, we generated SLC38A2-deficient B16-OVA (Cas9^+^) tumour cells (Extended Data Fig. 4g) and performed stable isotope tracing assays of glutamine and other known substrates of SLC38A2^36,37^. SLC38A2-deficient B16-OVA cells exhibited reduced uptake of ^13^C-labelled glutamine, and to a lesser extent alanine and serine (Extended Data Fig. 4h), indicating increased preferential glutamine transport by SLC38A2.

To establish the functional importance of SLC38A2 in tumour cells in mediating tumour–immune interactions, we challenged wild-type mice with sgNTC- or sg*Slc38a2*-transduced MC38 or B16-OVA cells, and found slower tumour growth upon SLC38A2 deletion (Fig. 2h and Extended Data Fig. 4i). Accordingly, abundance of glutamine, but not other SLC38A2 substrates, was increased in TIF from SLC38A2-deficient B16-OVA tumours (Extended Data Fig. 4j), further supporting that SLC38A2 more selectively mediates the transport of glutamine. Analysis of intratumoral lymphocytes in sg*Slc38a2*-transduced MC38 tumours revealed a higher frequency and number of intratumoral CD8^+^ T cells, including those expressing IFNγ, TNF or granzyme B (Fig. 2i,j and Extended Data Fig. 4k), indicative of enhanced effector phenotypes. To test whether SLC38A2 acts in a tumour-intrinsic manner or requires the immune system for its in vivo effects, we examined growth of SLC38A2-deficient MC38 tumours in *Batf3*^*–/–*^ or *Rag1*^*–/–*^ mice. The beneficial anti-tumour effect was blocked in these mice (Fig. 2k and Extended Data Fig. 4l), revealing the critical importance of cDC1s and lymphocytes. Collectively, these results show that SLC38A2 deficiency in tumour cells markedly impairs tumour growth in a cDC1-dependent manner.

## SLC38A2 in cDC1s triggers tumour immunity

**Extended Data Fig. 5:**
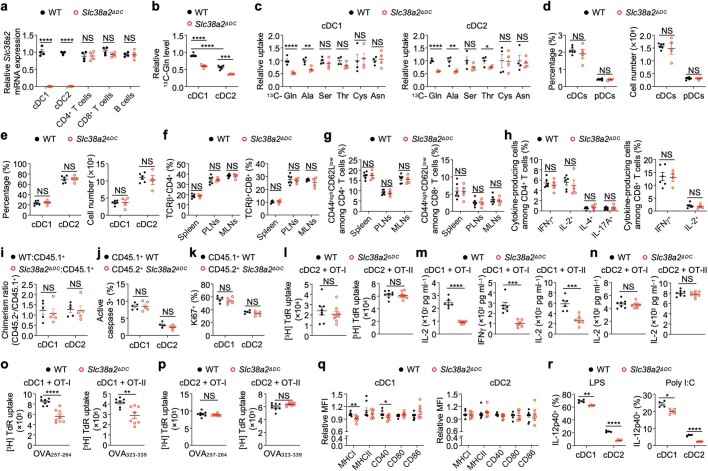
DC and T cell phenotypes in *Slc38a2*^*ΔDC*^ mice. **a**, Real-time PCR analysis of *Slc38a2* mRNA levels in different splenic immune cell populations from indicated mice (*n* = 4 per genotype). WT, wild-type. **b**, Intracellular ^13^C-glutamine (Gln) levels in wild-type or SLC38A2-deficient cDC1s (*n* = 5 each genotype) and cDC2s (*n* = 6 for wild-type; 4 for *Slc38a2*^*ΔDC*^) after 10 min of incubation. **c**, Intracellular levels of ^13^C-labelled amino acids in wild-type and SLC38A2-deficient splenic cDC1s and cDC2s after 10 min of incubation (*n* = 4 per genotype). Ala, alanine; Ser, serine; Thr, threonine; Cys, cysteine; Asn, asparagine. cDCs were sort-purified from wild-type and *Slc38a2*^*ΔDC*^ mice. **d**,**e**, Splenic cDCs and pDCs (**d**) or cDC1s and cDC2s (**e**) in wild-type (*n* = 6) and *Slc38a2*^*ΔDC*^ (*n* = 4) mice. **f**,**g**, Total (**f**) or CD44^high^CD62L^low^ (**g**) CD4^+^ and CD8^+^ T cells from wild-type (*n* = 6) and *Slc38a2*^*ΔDC*^ (*n* = 4) mice. PLNs, peripheral lymph nodes; MLNs, mesenteric lymph nodes. **h**, IFNγ-, IL-2-, IL-4- or IL-17A-producing CD4^+^, and IFNγ- or IL-2-producing CD8^+^ T cells from wild-type (*n* = 6) and *Slc38a2*^*ΔDC*^ (*n* = 4) mice. **i**, cDC1 and cDC2 chimerism ratios (normalized to internal B cells) in indicated mixed BM chimeras (*n* = 5 each group). **j**,**k**, Active caspase 3^+^ (**j**) or Ki67^+^ (**k**) cDC1s and cDC2s from *Slc38a2*^*ΔDC*^:CD45.1^+^ mixed BM chimeras (*n* = 5 each group). **l**, [^3^H] Thymidine (TdR) incorporation by OT-I and OT-II cells after coculture with wild-type or SLC38A2-deficient OVA-pulsed cDC2s (*n* = 8 per genotype). **m**,**n**, IL-2 or IFNγ production by OT-I or OT-II cells after coculture with OVA-pulsed wild-type or SLC38A2-deficient cDC1s (**m**) and cDC2s (**n**) as indicated (*n* = 6 per genotype for cDC1s, *n* = 8 per genotype for cDC2s). **o**,**p**, [^3^H] TdR incorporation by OT-I or OT-II cells after coculture with OVA_257–264_- or OVA_323–339_-pulsed WT or SLC38A2-deficient cDC1s (**o**) and cDC2s (**p**) as indicated (*n* = 8 per genotype) **q**, Expression of indicated molecules on splenic cDC1s and cDC2s from wild-type (*n* = 8) and *Slc38a2*^*ΔDC*^ (*n* = 7) mice. **r**, IL-12p40^+^ wild-type or SLC38A2-deficient cDC1s and cDC2s after lipopolysaccharide (LPS) or poly I:C stimulation (*n* = 4 per genotype). Data are means ± s.e.m. **a**–**r**, Two-tailed unpaired Student’s *t*-test. Data are representative of one (**c**), two (**a**,**b**,**i**–**k**,**r**) or three (**l**–**p**), or pooled from two (**d**–**h**, **q**) independent experiments. *P < 0.05, **P < 0.01, ***P < 0.001, ****P < 0.0001. NS, not significant. Source data

To further explore the relationship between SLC38A2, glutamine uptake and cDC1 function, we generated mice bearing DC-specific deletion of *Slc38a2* via *Cd11c*^*cre*^ mice (*Slc38a2*^*ΔDC*^), which resulted in decreased uptake of glutamine, and to a lesser extent alanine, by cDC1s and cDC2s (Extended Data Fig. 5a–c), revealing SLC38A2 as a critical glutamine transporter in DCs. Under steady state, *Slc38a2*^*ΔDC*^ mice exhibited normal homeostasis of DCs and T cells (Extended Data Fig. 5d–h). To explore whether SLC38A2 deficiency impairs the survival of DCs, we generated mixed bone marrow chimeras (Methods), and observed comparable chimerism ratios in cDC1s and cDC2s from *Slc38a2*^*ΔDC*^:CD45.1^+^ chimeras compared with controls (Extended Data Fig. 5i). Moreover, SLC38A2-deficient cDC1s and cDC2s had unaltered frequencies of active caspase-3^+^ and Ki67^+^ cells (Extended Data Fig. 5j,k). Thus, SLC38A2 has a role in glutamine transport in DCs but is dispensable for their homeostasis.

Notably, SLC38A2-deficient cDC1s, but not cDC2s, exhibited an impaired ability to promote T cell priming (Fig. 2l,m and Extended Data Fig. 5l–p). To examine the role of SLC38A2 in antigen presentation by cDC1s in vivo, we generated *Batf3*^–/–^:*Slc38a2*^*ΔDC*^ mixed bone marrow chimeras^16^ (and control mixed bone marrow chimeras;  Methods) to selectively restrict SLC38A2 deficiency to cDC1s. After adoptive transfer of OT-I cells and OVA immunization, OT-I cell proliferation was reduced to a similar extent in *Batf3*^–/–^:*Slc38a2*^*ΔDC*^ and *Slc38a2*^*ΔDC*^ chimeras (Fig. 2n), indicating a selective defect of cDC1s lacking SLC38A2. Accordingly, SLC38A2-deficient cDC1s showed modest reductions of CD40 and MHCI expression (Extended Data Fig. 5q). Further, IL-12p40 production was reduced in SLC38A2-deficient DCs (Extended Data Fig. 5r). Therefore, SLC38A2 expression in cDC1s is important for activating T cell immunity.

**Extended Data Fig. 6:**
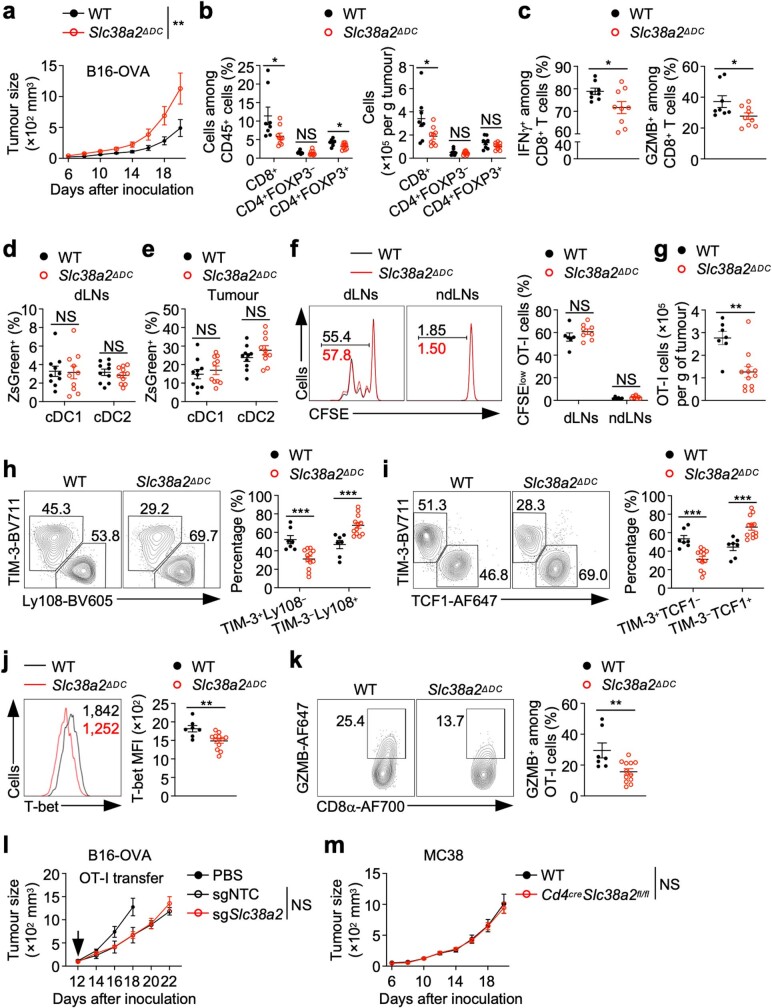
SLC38A2 deficiency in DCs impairs anti-tumour adaptive immunity. **a**, B16-OVA tumour growth in wild-type (*n* = 9) and *Slc38a2*^*∆DC*^ (*n* = 11) mice. WT, wild-type. **b**,**c**, Indicated T cell populations (**b**) or IFNγ^+^ or Granzyme B^+^ (GZMB^+^) CD8^+^ T cells (**c**) in MC38 tumours from wild-type (*n* = 8) and *Slc38a2*^*∆DC*^ (*n* = 9) mice at day 15. **d**,**e**, ZsGreen^+^ migratory cDC1s and cDC2s in tumour-draining lymph nodes (dLNs) (**d**) or tumours (**e**) from indicated mice bearing B16-ZsGreen tumours (*n* = 10 per genotype). **f**, CFSE^low^ OT-I cells in tumour dLNs or non-dLNs (ndLNs) of MC38-OVA tumour-bearing wild-type (*n* = 6) and *Slc38a2*^*∆DC*^ (*n* = 8) mice at day 2 after naïve OT-I cell adoptive transfer. **g**–**k**, Activated OT-I cells were transferred into B16-OVA tumour-bearing wild-type (*n* = 7) and *Slc38a2*^*∆DC*^ (*n* = 12) mice. OT-I cells were analysed at day 7 after adoptive transfer. OT-I cell number (**g**). Effector-like (TIM-3^+^Ly108^−^ or TIM-3^+^TCF1^−^) and stem-like (TIM-3^−^Ly108^+^ or TIM-3^−^TCF1^+^) (**h**,**i**) OT-I cells. T-bet mean fluorescence intensity (MFI) in OT-I cells (**j**). GZMB^+^ OT-I cells (**k**). **l**, B16-OVA tumour growth in mice receiving sgNTC- or sg*Slc38a2*-transduced OT-I cells at day 12 (*n* = 4 for sgNTC; 3 for sg*Slc38a2*; transfer indicated by an arrow). Non-transfer control mice (*n* = 3) received PBS. **m**, MC38 tumour growth in wild-type and *Cd4*^*cre*^*Slc38a2*^*fl/fl*^ mice (*n* = 7 per genotype). Data are means ± s.e.m. **b**–**g**,**j**,**k**, Two-tailed unpaired Student’s *t*-test. **a**,**h**,**i**,**l**,**m**, Two-way ANOVA. Data are representative of one (**d**–**k**) or two (**a**–**c**,**l**,**m**) independent experiments. *P < 0.05, **P < 0.01, ***P < 0.001. NS, not significant. Source data

Next, we challenged wild-type and *Slc38a2*^*ΔDC*^ mice with MC38 or B16-OVA cells and found increased tumour growth in *Slc38a2*^*ΔDC*^ mice (Fig. 2o and Extended Data Fig. 6a). However, intratumoral glutamine supplementation had little effect on MC38 tumour growth in *Slc38a2*^*ΔDC*^ mice (Fig. 2p), indicating a functional link between glutamine and SLC38A2 expressed by DCs in anti-tumour immunity. Accordingly, *Slc38a2*^*ΔDC*^ mice showed a decreased proportion and number of CD8^+^ T cells, including those expressing IFNγ and granzyme B, in the TME (Extended Data Fig. 6b,c). Upon challenge with MC38-OVA cells, *Slc38a2*^*ΔDC*^ mice had a reduced frequency and cellularity of intratumoral OVA-tetramer^+^CD8^+^ T cells, as well as decreased production of IFNγ and TNF upon stimulation with OVA_257–264_ (Fig. 2q,r), revealing a role of SLC38A2 in DCs in mediating antigen-specific CD8^+^ T cell responses.

Mechanistically, SLC38A2 deficiency did not impair antigen uptake or the migratory capacity of DCs, nor their functionality to induce naive T cell priming in tumour dLNs (Extended Data Fig. 6d–f). By contrast, intratumoral accumulation of adoptively transferred activated OT-I cells was reduced in B16-OVA-bearing *Slc38a*2^*ΔDC*^ mice (Extended Data Fig. 6g), associated with decreased proportions of effector-like cells and expression of T-bet and granzyme B in these OT-I cells (Extended Data Fig. 6h–k). Collectively, these results suggest that SLC38A2 expressed by DCs is essential for maintaining the effector function of antigen-specific CD8^+^ T cells in the TME.

Finally, to examine whether cDC1-specific expression of SLC38A2 is sufficient to control tumour growth, we generated *Xcr1*^*cre/+*^*Slc38a2*^*fl/fl*^ mice to delete SLC38A2 specifically in cDC1s^38^. MC38 tumour growth was increased in *Xcr1*^*cre*/+^*Slc38a2*^*fl/fl*^ mice (Fig. 2s), indicating the selective requirement of SLC38A2 in cDC1s for tumour control. By contrast, sgRNA-mediated deletion of *Slc38a2* in Cas9-expressing OT-I cells did not impair their ability to control B16-OVA tumour growth (Extended Data Fig. 6l). Similar results were obtained upon genetic deletion of *Slc38a2* in T cells (*Cd4*^*cre*^*Slc38a2*^*fl/fl*^ mice) in the MC38 tumour model (Extended Data Fig. 6m). Thus, DCs but not T cells require SLC38A2 to orchestrate anti-tumour immunity, suggesting that SLC38A2 represents a competitive checkpoint between tumour cells and cDC1s for glutamine acquisition and tumour–immune interactions.

## FLCN in cDC1s mediates tumour immunity

**Fig. 3:**
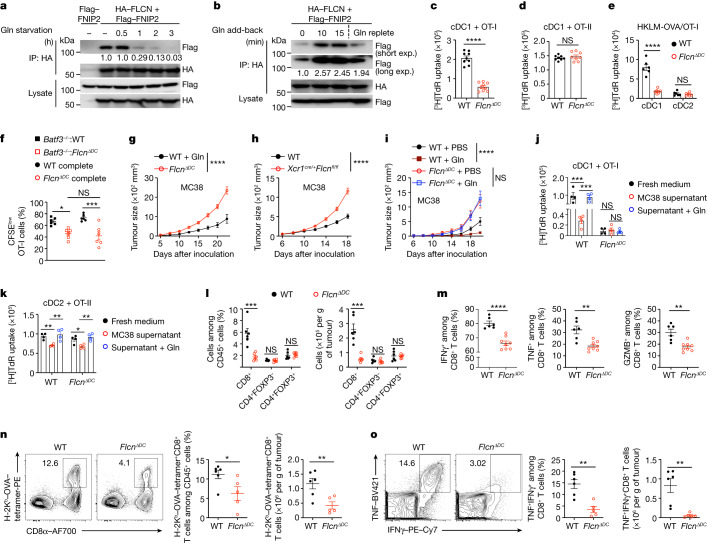
Glutamine promotes the priming effect and anti-tumour immunity of cDC1s via FLCN. **a**,**b**, The interaction of haemagglutinin (HA)–FLCN and Flag–FNIP2 in HEK293T cells cultured in glutamine-free medium (**a**) or after glutamine starvation and replacement (**b**) for the indicated time. Numbers indicate the relative intensity of the Flag band. Exp., exposure. **c**–**e**, [^3^H]Thymidine incorporation by OT-I (**c,** **e**) or OT-II (**d**) cells after coculture with OVA-pulsed (**c**,**d**) or HKLM-OVA-pulsed (**e**) splenic wild-type or FLCN-deficient cDC1s (**c**,**e**) or cDC2s (**d**,**e**) (*n* = 9 (**c**), *n* = 8 (**d**) or *n* = 6 (**e**) per genotype). **f**, CFSE^low^ OT-I cells in indicated OVA-immunized chimeric mice at day 3 after challenge (*n* = 6 per group). **g**,**h**, Growth of MC38 tumours in wild-type (*n* = 6) and *Flcn*^*ΔDC*^ (*n* = 5) mice (**g**) or wild-type (*n* = 10) and *Xcr1*^*cre/+*^*Flcn*^*fl/fl*^ (*n* = 8) mice (**h**). **i**, MC38 tumour growth in the indicated mice given intratumoral PBS or glutamine (*n* = 8 mice per group). **j**,**k**, [^3^H]Thymidine incorporation by OT-I (**j**) or OT-II (**k**) cells cocultured with wild-type and FLCN-deficient cDC1s (**j**) or cDC2s (**k**) pulsed with OVA in fresh medium, MC38 supernatant or MC38 supernatant plus glutamine (*n* = 4 per group). **l**, T cell populations in MC38 tumours from the indicated mice at day 15 (*n* = 6 per genotype). **m**, IFNγ^+^, TNF^+^ and granzyme B^+^ (GZMB^+^) CD8^+^ T cells in MC38 tumours from wild-type (*n* = 6) and *Flcn*^*ΔDC*^ (*n* = 8) mice at day 15. **n**,**o**, OVA-specific CD8^+^ T cells (**n**) and TNF^+^IFNγ^+^ CD8^+^ T cells (**o**) in MC38-OVA tumours from wild-type (*n* = 6) and *Flcn*^*ΔDC*^ (*n* = 5) mice at day 19. Data are mean ± s.e.m. **c**–**e**,**l**–**o**, Two-tailed unpaired Student’s *t*-test. **f**, One-way ANOVA. **g**–**k**, Two-way ANOVA. Data are representative of two (**a**,**b**,**e**,**f**,**j**,**k**,**n**,**o**) or at least three (**c**,**d**,**g**–**i**,**l**,**m**) independent experiments. *P < 0.05, **P < 0.01, ***P < 0.001, ****P < 0.0001. NS, not significant. Source data

**Extended Data Fig. 7:**
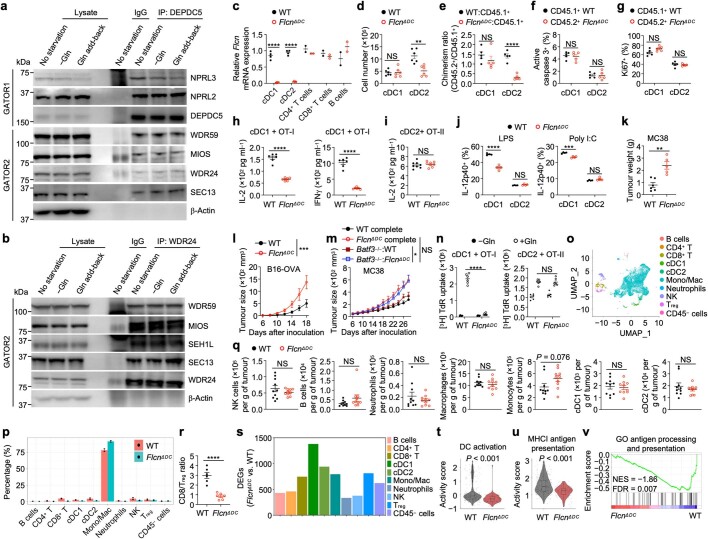
GATOR1−GATOR2 complex assembly or interaction is not influenced by glutamine, and FLCN deficiency in DCs impairs anti-tumour immunity. **a**,**b**, Immunoblot analysis of GATOR1 or GATOR2 complex proteins in glutamine (Gln)-starved HEK293T cells after Gln refeeding for 15 min (Gln add-back). No starvation, cells maintained in Gln-sufficient medium. Immunoprecipitation (IP) was performed using anti-DEPDC5 (**a**) or anti-WDR24 (**b**) antibody. kDa, kilodaltons. **c**, Real-time PCR analysis of *Flcn* mRNA levels in indicated cell populations from wild-type and *Flcn*^*∆DC*^ mice (*n* = 4 per genotype for cDC1s and cDC2s; 2 per genotype for other cell types). WT, wild-type. **d**, Number of splenic cDC1s and cDC2s from indicated mice (*n* = 6 per genotype). **e**, cDC1 and cDC2 chimerism ratios (normalized to internal B cells) in indicated mixed BM chimeras (*n* = 5 each group). **f**,**g**, Active caspase 3^+^ (**f**) or Ki67^+^ (**g**) cDC1s and cDC2s from *Flcn*^*ΔDC*^:CD45.1^+^ mixed BM chimeras (*n* = 5 each group). **h**,**i**, IL-2 (**h**,**i**) and IFNγ (**h**) production by OT-I (**h**) and OT-II (**i**) cells after coculture with OVA-pulsed splenic wild-type or FLCN-deficient cDC1s (**h**; *n* = 6 per genotype) or cDC2s (**i**; *n* = 8 per genotype). **j**, IL-12p40^+^ wild-type and FLCN-deficient cDC1s or cDC2s after lipopolysaccharide (LPS) or poly I:C stimulation (*n* = 4 per genotype). **k**, MC38 tumour weight (at day 20) in wild-type (*n* = 6) and *Flcn*^*∆DC*^ (*n* = 5) mice. **l**, Tumour growth curves in wild-type (*n* = 7) and *Flcn*^*∆DC*^ (*n* = 4) mice inoculated with B16-OVA cells. **m**, MC38 tumour growth in indicated chimeric mice (*n* = 8 for *Batf3*^−/−^:WT chimeras; 6 for other chimeras). **n**, [^3^H] Thymidine (TdR) incorporation by OT-I and OT-II cells after coculture with cDC1s or cDC2s pulsed with OVA in Gln-sufficient (+Gln) or Gln-free (−Gln) medium (*n* = 6 each group). **o**,**p**, Intratumoral CD45^+^ cells and cDCs were sorted from MC38 tumour-bearing wild-type and *Flcn*^*∆DC*^ mice at day 15 and mixed at ratio of 2:1 for scRNA-seq analysis. UMAP plots of cells indicated by cell clusters (**o**). Frequencies of indicated cell populations (*n* = 2 per genotype) (**p**). **q**, Analysis of indicated intratumoral immune cell populations in MC38 tumour-bearing wild-type and *Flcn*^*∆DC*^ mice (*n* = 10 per genotype). **r**, CD8^+^ T to T_reg_ cell ratio in MC38 tumours from indicated mice at day 15 (*n* = 6 per genotype). **s**, Bar graph showing the number of differentially expressed genes (DEGs; *Flcn*^*∆DC*^ versus wild-type) in each scRNA-seq cell cluster (**o**). **t**,**u**, Violin plots showing the activity scores of indicated gene signatures in intratumoral cDC1s (profiled by scRNA-seq) from PBS (*n* = 279 cells)- or Gln (*n* = 90 cells)-treated MC38 tumours. **v**, GSEA enrichment plot showing downregulation of indicated gene signature in intratumoral FLCN-deficient versus wild-type cDC1s (profiled by scRNA-seq). FDR, false discovery rate; NES, normalized enrichment score. Data are means ± s.e.m. **c**–**k**,**q**,**r**, Two-tailed unpaired Student’s *t*-test. **l**–**n**, Two-way ANOVA. **t**,**u**, Two-tailed Wilcoxon rank sum test. Data are representative of one (**m**), two (**a**–**g**,**j**,**q**) or at least three (**h**,**i**,**k**,**l**,**n**,**r**) independent experiments. *P < 0.05, **P < 0.01, ***P < 0.001, ****P < 0.0001. NS, not significant. Source data

Nutrient-responsive complexes—including GATOR1, GATOR2 and FLCN–FNIP—mediate amino acid-induced intracellular signalling^39,40^, although how specific amino acids modulate their activity is unclear in immune cells. We found that glutamine had no effect on the assembly of GATOR1 or GATOR2 complexes or the interaction between GATOR1 and GATOR2^39^ (Extended Data Fig. 7a,b). By contrast, FLCN−FNIP2 interaction was reduced upon glutamine starvation, and adding back glutamine promoted the formation of the FLCN–FNIP2 complex (Fig. 3a,b). Therefore, glutamine availability regulates the assembly of the FLCN–FNIP2 complex, but not GATOR1 or GATOR2 complexes.

Given these results, we tested the functional importance of FLCN in DC-mediated adaptive and anti-tumour immunity by generating *Flcn*^*ΔDC*^ (via *Cd11c*^*cre*^) mice and mixed bone marrow chimeras, which showed cell-intrinsic decreases in cDC2 but not cDC1 number in spleen under steady state conditions (Extended Data Fig. 7c–e). However, there were similar frequencies of active caspase-3^+^ and Ki67^+^ cDC1s and cDC2s from *Flcn*^*ΔDC*^:CD45.1^+^ mixed chimeras (Extended Data Fig. 7f,g), suggesting that FLCN contributes to cDC2 development rather than survival or proliferation. We next assessed the function of FLCN-deficient DCs, and found that FLCN-deficient cDC1s but not cDC2s showed a pronounced defect in driving T cell priming (Fig. 3c,d and Extended Data Fig. 7h,i). Further, FLCN-deficient cDC1s were defective in mediating HKLM-OVA-induced T cell proliferation (Fig. 3e). Associated with these functional defects, FLCN-deficient cDC1s produced less IL-12p40 (Extended Data Fig. 7j). Further, OT-I cell proliferation in vivo was reduced to a similar extent in OVA-immunized *Batf3*^–/–^:*Flcn*^*ΔDC*^ mixed and *Flcn*^*ΔDC*^ complete bone marrow chimeras^16^ (Fig. 3f), indicating a selective defect of cDC1s lacking FLCN. Consistent with these functional defects, the growth of MC38 or B16-OVA tumours was increased in *Flcn*^*ΔDC*^ mice (Fig. 3g and Extended Data Fig. 7k,l), indicating a crucial role of FLCN in DCs in restricting tumour growth. These effects were attributed to FLCN deletion selectively in cDC1s, as evidenced by increased MC38 tumour growth in *Batf3*^*–/–*^:*Flcn*^*ΔDC*^ mixed chimeras or *Xcr1*^*cre/+*^*Flcn*^*fl/fl*^ mice with cDC1-specific FLCN deletion (Fig. 3h and Extended Data Fig. 7m).

Of note, FLCN deletion in DCs rendered mice unresponsive to the beneficial anti-tumour effect of glutamine supplementation (Fig. 3i), indicating a requirement for FLCN expression in DCs in mediating this therapeutic effect. To determine whether FLCN is required for glutamine in promoting cDC1 function, we tested whether FLCN deletion affects the ability of cDC1s or cDC2s to drive glutamine-dependent T cell priming. FLCN was essential for mediating the responsiveness of cDC1s but not cDC2s to glutamine in promoting T cell proliferation (Extended Data Fig. 7n). Further, unlike wild-type cDC1s, FLCN-deficient cDC1 function was not further affected by MC38 supernatant with or without glutamine supplementation, whereas FLCN-deficient cDC2s remained responsive to these treatments (Fig. 3j,k). In sum, FLCN is selectively required for glutamine to promote the function of cDC1s but not cDC2s, concomitant with its critical importance in vivo in mediating glutamine-dependent suppression of tumour growth.

To determine the mechanistic basis of this effect, we profiled intratumoral CD45^+^ cells in wild-type and *Flcn*^*ΔDC*^ mice challenged with MC38 tumour cells using scRNA-seq (Extended Data Fig. 7o). Intratumoral CD8^+^ T cells were selectively decreased among CD45^+^ immune cells; this effect was validated by flow cytometry analysis (Fig. 3l and Extended Data Fig. 7p,q). The ratio of CD8^+^ T cells to T_reg_ cells was also decreased (Extended Data Fig. 7r), indicative of a more immunosuppressive TME. Additionally, cDC1s from *Flcn*^*ΔDC*^ mice bearing MC38 tumours had the largest number of differentially expressed genes (Extended Data Fig. 7s), indicating a pronounced effect of FLCN deficiency on cDC1s in the TME. Further, FLCN-deficient cDC1s had lower activity scores of gene signatures related to DC activation and MHCI antigen presentation (Extended Data Fig. 7t,u), as well as downregulated antigen processing and presentation pathways (Extended Data Fig. 7v) assessed by GSEA, in line with the defective antigen presentation capacity of FLCN-deficient cDC1s. Thus, FLCN loss leads to impaired DC functional fitness, associated with reduced CD8^+^ T cell accumulation in the TME.

**Extended Data Fig. 8:**
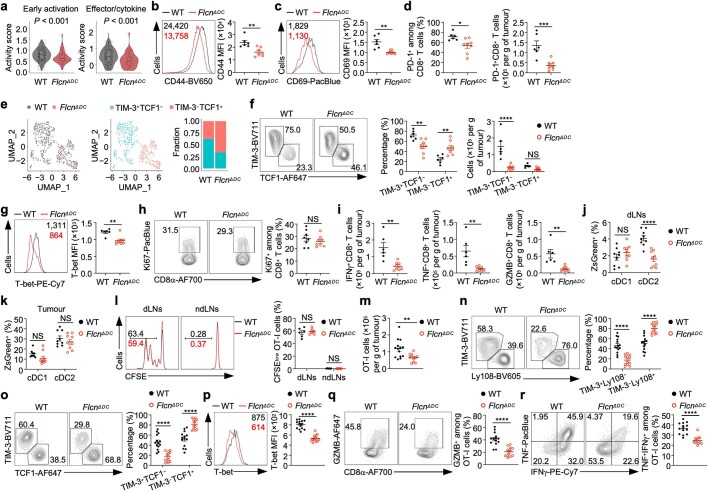
FLCN deficiency in DCs impairs effector function of intratumoral CD8^+^ T cells. **a**, Violin plots showing activity scores of indicated gene signatures in intratumoral CD8^+^ T cells (profiled by scRNA-seq). **b**,**c**, CD44 (**b**) and CD69 (**c**) expression on CD8^+^ T cells in MC38 tumours from wild-type (*n* = 6) and *Flcn*^*∆DC*^ (*n* = 7) mice at day 15. MFI, mean fluorescence intensity; WT, wild-type. **d**, PD-1^+^CD8^+^ T cells in MC38 tumours from wild-type (*n* = 6) and *Flcn*^*∆DC*^ (*n* = 8) mice at day 15. **e**, UMAP plots showing distributions of effector-like (TIM-3^+^TCF1^−^) and stem-like (TIM-3^−^TCF1^+^) cells among intratumoral CD8^+^ T cells from indicated MC38 tumour-bearing mice. Right, indicated intratumoral CD8^+^ T cell subclusters quantification. **f**,**g**, TIM-3^+^TCF1^−^ and TIM-3^−^TCF1^+^ CD8^+^ T cells (f) and T-bet MFI in intratumoral CD8^+^ T cells (g) in MC38 tumours from wild-type (*n* = 6) and *Flcn*^*∆DC*^ (*n* = 8) mice at day 15. **h**, Ki67^+^ CD8^+^ T cells in MC38 tumours from indicated mice (*n* = 8 per genotype). **i**, IFNγ^+^, TNF^+^ or Granzyme B^+^ (GZMB^+^) CD8^+^ T cells from mice as in **d**,**f**. **j**,**k**, ZsGreen^+^ migratory cDC1s and cDC2s in tumour-draining lymph nodes (dLNs; **j**) or tumours (**k**) from indicated B16-ZsGreen tumour-bearing mice at day 16 (*n* = 9 per genotype). **l**, CFSE^low^ OT-I cells in tumour dLNs and non-dLNs (ndLNs) of indicated MC38-OVA tumour-bearing mice (*n* = 6 per genotype) at day 2 after naïve OT-I cell transfer. **m**–**r**, Activated OT-I cells were transferred into B16-OVA tumour-bearing wild-type (*n* = 14) and *Flcn*^*∆DC*^ (*n* = 12) mice. OT-I cells were analysed at day 7 after adoptive transfer. OT-I cell number (**m**). TIM-3^+^Ly108^−^ and TIM-3^−^Ly108^+^ (**n**) or TIM-3^+^TCF1^−^ and TIM-3^−^TCF1^+^ (**o**) OT-I cells. T-bet in OT-I cells (**p**). GZMB^+^ (**q**) or TNF^+^IFNγ^+^ (**r**) OT-I cells. Data are means ± s.e.m. **a**, Two-tailed Wilcoxon rank sum test. **b**–**d**,**g**–**m**,**p**–**r**, Two-tailed unpaired Student’s *t*-test. **f**,**n**,**o**, Two-way ANOVA. Data are representative of one (**j**–**r**), two (**c**,**g**,**h**) or at least three (**b**,**d**,**f**,**i**) independent experiments. *P < 0.05, **P < 0.01, ***P < 0.001, ****P < 0.0001. NS, not significant. Source data

Consistent with DC functional defects, intratumoral CD8^+^ T cells from *Flcn*^*ΔDC*^ mice showed reduced activity scores of gene signatures associated with CD8^+^ T cell early activation and effector/cytokine signalling (Extended Data Fig. 8a). Further, intratumoral CD8^+^ T cells from *Flcn*^*ΔDC*^ mice had reduced expression of CD44, CD69 and T-bet, as well as decreased numbers of PD-1^+^ and TIM-3^+^TCF1^–^ cells without alteration of Ki67 expression (Extended Data Fig. 8b–h). Consistent with these changes, IFNγ^+^, TNF^+^ and granzyme B^+^ CD8^+^ T cells were decreased in tumours from *Flcn*^*ΔDC*^ mice (Fig. 3m and Extended Data Fig. 8i), suggesting impaired activation and effector function. Indeed, there were fewer antigen-specific CD8^+^ T cells in MC38-OVA tumours from *Flcn*^*ΔDC*^ mice (Fig. 3n). Moreover, intratumoral CD8^+^ T cells exhibited impaired production of IFNγ and TNF upon stimulation with OVA_257–264_ (Fig. 3o). Together, these results indicate that FLCN is essential for cDC1s to prime anti-tumour CD8^+^ T cell effector responses.

Analysis of B16-ZsGreen tumour-bearing mice indicated that FLCN-deficient cDC2s but not cDC1s exhibited migratory defects to the tumour dLNs without alterations of antigen uptake by DCs (Extended Data Fig. 8j,k). Although there was no effect on naive OT-I cell priming in tumour dLNs, there was reduced accumulation of adoptively transferred activated OT-I cells and effector-like cells in the TME from *Flcn*^*ΔDC*^ mice (Extended Data Fig. 8l–o). Accordingly, these OT-I cells showed decreased expression of effector molecules (Extended Data Fig. 8p–r). Thus, FLCN expression in DCs is required primarily for the activation and effector function of cytotoxic CD8^+^ T cells in the TME, but not for naive CD8^+^ T cell priming in the tumour dLNs or for cDC1 migration.

## The glutamine–FLCN axis impedes TFEB in cDC1s

**Fig. 4:**
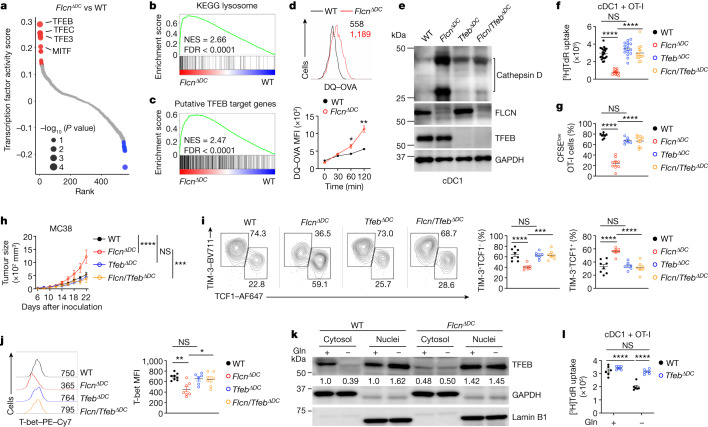
Co-deletion of TFEB restores the priming effect of FLCN-deficient or glutamine-deprived cDC1s. **a**, Transcription factor footprinting analysis of ATAC-seq peaks in wild-type and FLCN-deficient splenic cDC1s, ranked by activity score. Selected transcription factors are indicated. Red and blue dots depict upregulated and downregulated activities of transcription factors, respectively, with select transcription factors indicated. **b**,**c**, GSEA enrichment plots showing upregulation of KEGG lysosome pathway (**b**) and putative TFEB target genes in FLCN-deficient cDC1s (**c**). FDR, false discovery rate; NES, normalized enrichment score. **d**, Wild-type and FLCN-deficient splenic cDC1s (*n* = 3 per genotype) were incubated with DQ-OVA for the indicated times in DQ-OVA degradation assays. Numbers indicate mean fluorescence intensity (MFI) of DQ-OVA. **e**, Immunoblot analysis of cathepsin D (pro and mature forms), FLCN, TFEB and GAPDH expression in cDC1s from the indicated mice. **f**, [^3^H]Thymidine incorporation by OT-I cells after coculture with splenic cDC1s from wild-type (*n* = 21), *Flcn*^*ΔDC*^ (*n* = 12), *Tfeb*^*ΔDC*^ (*n* = 15) and *Flcn/Tfeb*^*ΔDC*^ (*n* = 12) mice. **g**, CFSE^low^ OT-I cells in OVA-immunized wild-type (*n* = 8), *Flcn*^*ΔDC*^ (*n* = 9), *Tfeb*^*ΔDC*^ (*n* = 6) or *Flcn/Tfeb*^*ΔDC*^ (*n* = 10) mice on day 3 after challenge. **h**, MC38 tumour growth in wild-type (*n* = 9), *Flcn*^*ΔDC*^ (*n* = 7), *Tfeb*^*ΔDC*^ (*n* = 9) and *Flcn/Tfeb*^*ΔDC*^ (*n* = 7) mice. **i**,**j**, Effector-like (TIM-3^+^TCF1^−^) or stem-like (TIM-3^−^TCF1^+^) subsets (**i**) and MFI of T-bet (**j**) in CD8^+^ T cells in MC38 tumours from wild-type (*n* = 8), *Flcn*^*ΔDC*^ (*n* = 7), *Tfeb*^*ΔDC*^ (*n* = 6) and *Flcn/Tfeb*^*ΔDC*^ (*n* = 7) mice. Graph in **j** shows histogram of T-bet, with quantified MFI indicated. **k**, TFEB protein levels in cytosolic and nuclear fractions in wild-type and FLCN-deficient splenic cDC1s. Numbers indicate TFEB abundance in cytosolic or nuclear TFEB (normalized to GAPDH or lamin B, respectively) versus those in wild-type cDC1s treated with glutamine. **l**, [^3^H]Thymidine incorporation by OT-I cells after coculture with wild-type and TFEB-deficient splenic cDC1s pulsed with OVA in glutamine-replete or glutamine-deficient medium (*n* = 6 per genotype). Data are mean ± s.e.m. **a**,**d**, Two-tailed unpaired Student’s *t*-test. **f**,**g**,**i**,**j**,**l**, One-way ANOVA. **h**, Two-way ANOVA. Data are representative of two (**d**,**e**,**j**,**l**) or at least three (**h**,**i**,**k**) independent experiments or pooled from two (**g**) or three (**f**) independent experiments. *P < 0.05, **P < 0.01, ***P < 0.001, ****P < 0.0001. NS, not significant. Source data

**Extended Data Fig. 9:**
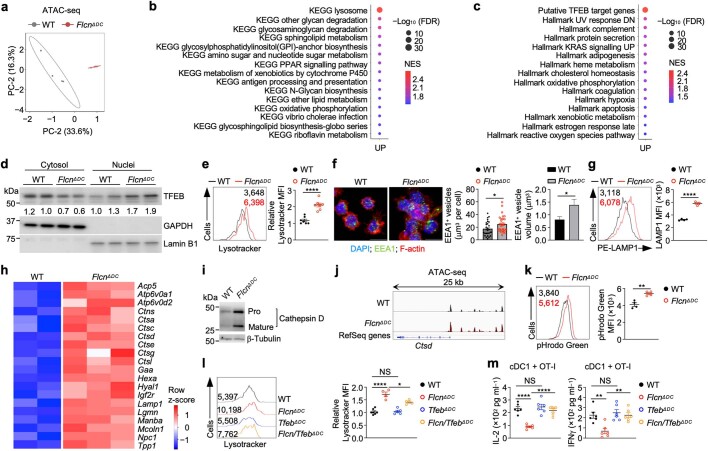
Enhanced lysosomal activation in FLCN-deficient cDC1s is rescued by TFEB co-deletion. **a**, Principal component analysis (PCA) plot of ATAC-seq data for wild-type and FLCN-deficient cDC1s. Percentage of variance is shown. WT, wild-type. **b**,**c**, GSEA of transcriptome analysis of FLCN-deficient versus wild-type cDC1s using KEGG pathway (**b**) and Hallmark gene sets combined with putative TFEB target genes (**c**). False discovery rate (FDR) and normalized enrichment score (NES) of top 15 pathways (ranked by NES) were visualized. **d**, TFEB protein levels in cytosolic and nuclear fractions in wild-type and FLCN-deficient splenic cDC1s. Numbers indicate TFEB abundance in cytosolic or nuclear TFEB (normalized to GAPDH or Lamin B1, respectively) versus wild-type. kDa, kilodaltons. **e**, Flow cytometry analysis (left) and quantification (right) of Lysotracker staining in splenic cDC1s from wild-type (*n* = 7) and *Flcn*^*ΔDC*^ (*n* = 7) mice. MFI, mean fluorescence intensity. **f**, Imaging analysis of volume of EEA1^+^ vesicles per cell (*n* = 28 for wild-type; 29 for *Flcn*^*ΔDC*^) or an individual EEA1^+^ vesicle (*n* = 496 for wild-type; 512 for *Flcn*^*ΔDC*^) in wild-type or FLCN-deficient cDC1s. Scale bar, 10 μm. **g**, MFI of LAMP1 in wild-type (*n* = 4) or FLCN-deficient (*n* = 3) splenic cDC1s. **h**, Heatmap showing expression of top 20 differentially expressed genes (ranked by Log_2_FC) from KEGG lysosome pathway in FLCN-deficient (*n* = 3) versus wild-type (*n* = 2) splenic cDC1s. **i**, Cathepsin D (pro and mature forms) and β-Tubulin expression in wild-type and FLCN-deficient cDC1s. **j**, Accessibility of the *Ctsd* gene and its upstream regions in wild-type and FLCN-deficient cDC1s assessed by ATAC-seq. **k**, pHrodo Green Dextran MFI in splenic wild-type and FLCN-deficient cDC1s (*n* = 4 per genotype). **l**, MFI of Lysotracker in splenic cDC1s from wild-type (*n* = 7), *Flcn*^*ΔDC*^ (*n* = 4), *Tfeb*^*ΔDC*^(*n* = 5) and *Flcn/Tfeb*^*ΔDC*^ (*n* = 4) mice. **m**, IL-2 and IFNγ production by OT-I cells after coculture with OVA-pulsed splenic cDC1s from the indicated mice (*n* = 6 per genotype). Data are means ± s.e.m. **e**–**g**,**k**, Two-tailed unpaired Student’s *t*-test. **l**,**m**, One-way ANOVA. Data are representative of two (**d**,**f**,**g**,**i**,**k**), or three (**m**), or pooled from three (**e**,**l**) independent experiments. *P < 0.05, **P < 0.01, ****P < 0.0001. NS, not significant. Source data

To establish the molecular mechanisms, we performed assay for transposase-accessible chromatin with high-throughput sequencing (ATAC-seq) using wild-type and FLCN-deficient cDC1s, followed by chromatin accessibility and transcription factor footprinting activity analyses. FLCN-deficient cDC1s exhibited an altered chromatin state, with enhanced activity of the microphthalmia (MiT/TFE) family of transcription factors, specifically TFEB and TFE3 (Fig. 4a and Extended Data Fig. 9a). TFEB and TFE3 are nutrient- and stress-sensitive transcription factors that orchestrate lysosomal biogenesis and function^41^. Accordingly, transcriptome profiling and GSEA showed that the lysosome pathway was upregulated in FLCN-deficient cDC1s (Fig. 4b, Extended Data Fig. 9b and Supplementary Table 2). Further, FLCN-deficient cDC1s were enriched for a curated TFEB gene target signature (Fig. 4c, Extended Data Fig. 9c and Supplementary Table 2). Consistent with these results, FLCN-deficient cDC1s showed increased TFEB nuclear localization as well as enhanced lysosomal mass, early endosome volume and LAMP1 staining (Extended Data Fig. 9d–g), suggesting dysregulated endolysosomal homeostasis that may contribute to defective antigen presentation and T cell priming^42^. Further, several lysosomal cathepsins, including cathepsin D, were highly upregulated in FLCN-deficient cDC1s, associated with increased accessibility at *Ctsd* (Extended Data Fig. 9h–j). Accordingly, lysosome acidification and antigen degradation were elevated in FLCN-deficient cDC1s (Fig. 4d and Extended Data Fig. 9k). These results collectively indicate upregulated TFEB activity and lysosomal function upon FLCN deletion in cDC1s.

**Extended Data Fig. 10:**
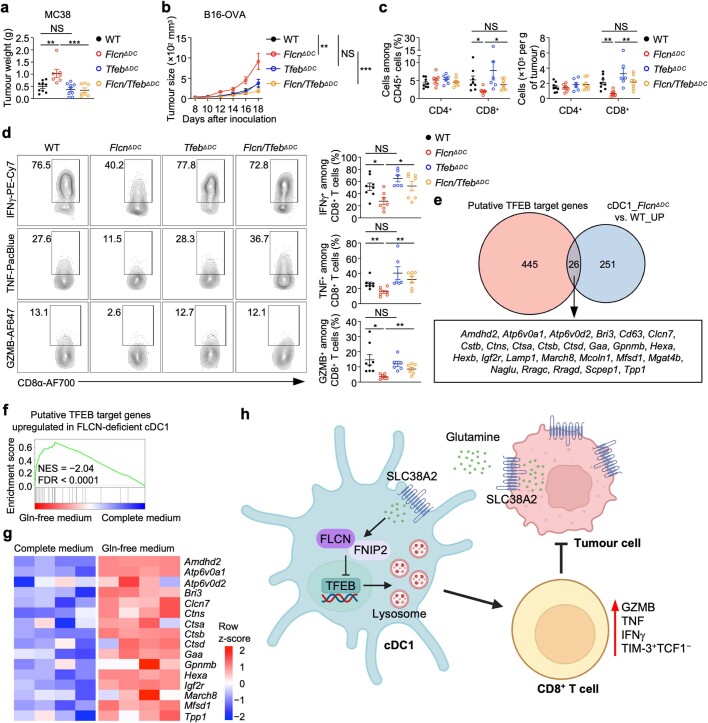
Roles of FLCN–TFEB signalling axis in mediating anti-tumour immunity and glutamine availability. **a**, MC38 tumour weight (at day 22) in wild-type (*n* = 9), *Flcn*^*ΔDC*^ (*n* = 7), *Tfeb*^*ΔDC*^ (*n* = 9) and *Flcn/Tfeb*^*ΔDC*^(*n* = 7) mice. WT, wild-type. **b**, B16-OVA tumour growth in wild-type (*n* = 11), *Flcn*^*ΔDC*^ (*n* = 11), *Tfeb*^*ΔDC*^ (*n* = 8) and *Flcn/Tfeb*^*ΔDC*^ (*n* = 7) mice. **c**, **d**, Total CD4^+^ and CD8^+^ T cells (**c**) or IFNγ^+^, TNF^+^ and Granzyme B^+^ (GZMB^+^) CD8^+^ T cells (**d**) in MC38 tumours from wild-type (*n* = 8), *Flcn*^*ΔDC*^ (*n* = 7), *Tfeb*^*ΔDC*^ (*n* = 6) and *Flcn/Tfeb*^*ΔDC*^ (*n* = 7) mice at day 15. **e**, Overlap of putative TFEB target genes and significantly upregulated genes in FLCN-deficient (versus wild-type) cDC1s. **f**, GSEA plot depicting the enrichment of gene set containing the 26 overlapped genes identified in **e**, in cDC1s treated with glutamine (Gln)-free medium versus complete medium. **g**, Heatmap showing expression of the leading-edge genes from Extended Data Fig. 10f in cDC1s treated with Gln-free medium and complete medium (*n* = 4 per group). **h**, Schematic of glutamine intercellular crosstalk between cDC1s and tumour cells, and nutrient signalling in cDC1s in modulating anti-tumour immunity. cDC1s and tumour cells both express SLC38A2 to mediate glutamine uptake to tune anti-tumour immunity, with tumour cells expressing higher levels of SLC38A2 than cDC1s. In cDC1s, glutamine induces FLCN–FNIP2 complex assembly and inhibits TFEB activity to promote the antigen presentation capacity of cDC1s. Consequently, glutamine-dependent signalling in cDC1s enhances cytokine production and generation of cytotoxic effector-like CD8^+^ T cells in the TME. The figure was generated using BioRender. Data are means ± s.e.m. **d**, Two-tailed unpaired Student’s *t*-test. **a**,**c**, One-way ANOVA. **b**, Two-way ANOVA. Data are representative of two (**a**–**d**) independent experiments. *P < 0.05, **P < 0.01, ***P < 0.001. NS, not significant. Source data

To establish the functional link between FLCN and TFEB, we generated *Tfeb*^*ΔDC*^ and *Flcn*^*ΔDC*^*Tfeb*^*ΔDC*^ (hereafter referred to as *Flcn/Tfeb*^*ΔDC*^) mice via the *Cd11c*^*cre*^ deletion system. Whereas TFEB deletion alone did not affect cathepsin D expression or lysosomal mass, co-deletion of TFEB and FLCN largely reversed the increased lysosomal mass and enhanced expression and maturation of cathepsin D in FLCN-deficient cDC1s (Fig. 4e and Extended Data Fig. 9l). TFEB co-deletion also restored the defect of FLCN-deficient cDC1s in mediating OT-I cell priming in vitro and in vivo (Fig. 4f,g and Extended Data Fig. 9m). Finally, TFEB co-deletion restored the defects of *Flcn*^*ΔDC*^ mice in controlling tumour growth, which were associated with a reversal of CD8^+^ T cell accumulation defects (Fig. 4h and Extended Data Fig. 10a–c). TFEB co-deletion also rescued the impaired generation of CD8^+^ T cells with effector-like phenotypes from *Flcn*^*ΔDC*^ mice (Fig. 4i,j and Extended Data Fig. 10d). Therefore, the FLCN–TFEB axis orchestrates cDC1 function and DC-mediated coordination of CD8^+^ T cell accumulation and function in the TME.

We hypothesized that glutamine availability shapes TFEB activity in an FLCN-dependent manner in cDC1s. Indeed, GSEA of cDC1s treated with glutamine-free or complete medium revealed enrichment of the curated gene set containing TFEB and FLCN-coregulated genes (Methods) in cDC1s treated with glutamine-free medium (Extended Data Fig. 10e,f), with lysosomal cathepsin genes *Ctsa*, *Ctsb* and *Ctsd* among the leading-edge genes (Extended Data Fig. 10g). Further, glutamine deprivation resulted in increased TFEB nuclear translocation in wild-type cDC1s but not in FLCN-deficient cDC1s (Fig. 4k), suggesting that glutamine restricts TFEB activity in an FLCN-dependent manner. In terms of cDC1 functional effects, TFEB-deficient cDC1s were largely resistant to glutamine deprivation-induced functional impairment in T cell priming (Fig. 4l), indicating the dependence of TFEB for mediating such impairment. These findings collectively reveal the critical role of FLCN–TFEB axis in orchestrating glutamine signalling and functional effects in cDC1s.

Nutritional alterations regulate immunity in infectious diseases and cancer^43^, with recent studies revealing the interplay between nutrients, tumour cells and immune cells in shaping tumour growth and anti-tumour immunity^6^. Whether and how nutrients affect DC subsets or functionality in anti-tumour immunity are largely unknown. Here, we found that intratumoral glutamine supplementation enhances anti-tumour immunity and immunotherapy efficacy by restoring the antigen presentation capacity of cDC1s. Our study also indicates that checkpoint blockade combined with glutamine supplementation represents a potential therapeutic strategy to overcome treatment resistance in patients with poor response to ICB therapy. Tumour cells and cDC1s rely on the glutamine transporter SLC38A2 for glutamine uptake and downstream biological effects. Of note, SLC38A2 more selectively mediates glutamine transport compared with its other substrates in these cells. Further, SLC38A2 expression in tumour cells restricts the access of cDC1s to glutamine, consistent with high glutamine uptake by tumour cells in the TME^7^. Notably, we establish that SLC38A2 expression in tumour cells and cDC1s has reciprocal effects on tumour growth, establishing SLC38A2-mediated glutamine acquisition as an intercellular metabolic checkpoint for controlling anti-tumour immunity. These findings advance our understanding of the signals driving DC activation by highlighting the roles of macronutrients as non-canonical signals that license DC function in the TME.

Glutamine has received broad attention for its metabolic effects in cancer cells^7,44^, T cells^21–23,45^ and macrophages^46^. How glutamine affects intracellular signalling events remains incompletely defined. In this Article, we show that glutamine availability reciprocally affects FLCN–FNIP2 complex formation and TFEB activity, and that the FLCN–TFEB axis mediates the glutamine effect on cDC1 antigen presentation and activation of effector-like CD8^+^ T cell responses (Extended Data Fig. 10h). Although FLCN suppresses TFEB activity^47^, we establish a previously unrecognized link between glutamine and the FLCN–TFEB signalling pathway in cDC1s and anti-tumour immunity in vivo. Notably, similar to FLCN deficiency, SLC38A2 deficiency in DCs eliminates the anti-tumour effect of glutamine supplementation, thereby establishing this glutamine transporter and FLCN signalling as critical drivers of cDC1 function and tumour immunity. Our work provides important insights into the immunostimulatory effects of glutamine in DCs, which contrast with the immunosuppressive or tolerogenic effects of lipids and indoleamine 2,3-dioxygenase 1-mediated tryptophan catabolism in modulating DC functions in tumours or inflammatory settings^48–50^. Collectively, targeting glutamine levels in tumours or glutamine-dependent signalling in cDC1s has implications for improving cancer treatments and overcoming tumour-mediated immunosuppression.

## Methods

### Mice

The research conducted in this study complies with all of the relevant ethical regulations. The animal protocols were approved by and performed in accordance with the Institutional Animal Care and Use Committee (IACUC) of St. Jude Children’s Research Hospital. Mice were housed and bred in specific pathogen-free conditions in the Animal Resource Center at St. Jude Children’s Research Hospital. Mice were on 12-hour light–dark cycles that coincide with daylight in Memphis, TN, USA. The St. Jude Children’s Research Hospital Animal Resource Center housing facility was maintained at 20–25 °C and 30–70% humidity. C57BL/6, CD45.1^+^, OT-I, OT-II, *Rag1*^−/−^, *Rosa*-*Cas9* knock-in^51^, *Batf3*^−/−^ (ref. ^29^), *Cd4*^*cre*^ (ref. ^52^), *Cd11c*^*cre*^ (ref. ^53^) and *Xcr1*^*cre*^ (ref. ^38^) mice were purchased from The Jackson Laboratory or as described previously^54^. *Slc38a2*^*fl/fl*^ mice were purchased from INFRAFRONTIER/EMMA. *Flcn*^*fl/fl*^ mice were kindly provided by L. Schmidt^55^. *Tfeb*^*fl/fl*^ mice were kindly provided by A. Ballabio^56^. The mice were backcrossed to the C57BL/6 background; sex- and age-matched mice were used throughout the study at 7–12 weeks old, and both male and female mice were used. The genetically modified mice were viable and developed normally. To generate mixed bone marrow chimeras, bone marrow cells from wild-type, *Slc38a2*^*ΔDC*^ or *Flcn*^*ΔDC*^ mice were mixed with cells from *Batf3*^−/−^ mice at a 1:1 ratio and transferred into lethally irradiated (11 Gy) CD45.1^+^ mice, followed by reconstitution for 6–8 weeks^16^. In certain experiments, bone marrow cells from wild-type or *Flcn*^*ΔDC*^ mice were transferred into lethally irradiated (11 Gy) CD45.1^+^ mice. For chimeras used in Extended Data Figs. 5i–k and 7e–g, bone marrow cells from wild-type, *Slc38a2*^*ΔDC*^ or *Flcn*^*ΔDC*^ mice were mixed with cells from CD45.1^+^ mice at a 1:1 ratio and transferred into lethally irradiated (11 Gy) C57BL/6 mice and analysed 8 weeks later. Age- and sex-matched mice with pre-determined genotypes (not blinded to investigators) were randomly assigned to control and experimental groups.

### Cell purification and culture

Mouse spleens were digested with 1 mg ml^−1^ collagenase IV (LS004188, Worthington) plus 200 U ml^−1^ DNase I (DN25, Sigma) for 45 min at 37 °C, and CD11c^+^ DCs were enriched using CD11c MicroBeads (130-125-835, Miltenyi Biotec) according to the manufacturer’s instructions. Enriched cells were stained and sorted for cDC1s (CD11c^+^CD8α^+^CD24^+^TCRβ^−^CD49b^−^B220^−^) and cDC2s (CD11c^+^MHCII^+^CD8α^−^CD11b^+^TCRβ^−^CD49b^−^B220^−^F4/80^−^Ly6C^−^) on a MoFlow (Beckman Coulter) or Reflection (i-Cyt) cell sorter. Lymphocytes from spleen and peripheral lymph nodes were sorted for naive OT-I cells (CD8^+^CD62L^high^CD44^low^CD25^−^) and naive OT-II cells (CD4^+^CD62L^high^CD44^low^CD25^−^) from OT-I and OT-II mice, respectively. Sorted DCs were cultured with specific medium as indicated in figure legends. Medium with or without individual amino acids was generated with RPMI 1640 powder (R8999-04A, US Biological) by supplementation of individual amino acids. The medium was supplemented with 10% (v/v) dialysed fetal bovine serum (FBS; A3382001, Thermo Fisher Scientific). For preparation of tumour cell line-derived culture supernatant, MC38 or B16F10 cells were cultured in glutamine-free RPMI 1640 medium (15-040-CV, Corning) supplemented with 10% (v/v) dialysed FBS plus 1% (v/v) penicillin-streptomycin (15140122, Thermo Fisher Scientific), and different concentrations of glutamine (0.3, 0.6 or 2 mM; 25030081, Thermo Fisher Scientific) as indicated in the figures. Tumour cell culture supernatant was collected 48 h later.

### In vitro BMDC culture

Bone marrow cells were flushed from mouse tibias and femurs, and red blood cells were lysed using ACK lysis buffer. Cells were then plated in RPMI 1640 medium supplemented with 10% (v/v) FBS, 1% (v/v) penicillin-streptomycin and 55 μM β-mercaptoethanol (RPMI 1640 complete medium). FLT3L BMDCs were cultured as previously described^16^. In brief, bone marrow cells were cultured in RPMI 1640 complete medium with 200 ng ml^−1^ FLT3L-Ig (BE0098, Bio X Cell) for 7–9 days. FLT3L BMDCs were sorted as cDC1s (B220^−^CD11c^+^CD24^+^CD172α^−^) and cDC2s (B220^−^CD11c^+^CD24^−^CD172α^+^) for further experiments. iCD103^+^ BMDCs were generated as previously described for Transwell assays^57^. In brief, bone marrow cells were plated in RPMI 1640 complete medium supplemented with 200 ng ml^−1^ FLT3L-Ig and 2 ng ml^−1^ mGM-CSF (315-03, Peprotech). Half of the fresh medium was supplemented to the cultures at day 5, and non-adherent cells were collected and replated in fresh medium at day 9. Loosely adherent cells were collected at days 15–17 for Transwell assays.

### Flow cytometry

For analysis of surface markers, cells were first incubated with Fc block (2.4G2, Bio X Cell) for 10 min in phosphate-buffered saline (PBS) containing 2% (w/v) FBS, and then stained with the appropriate antibodies on ice for appropriate 30 min. For intracellular cytokine detection, cells were stimulated for 4 h with phorbol 12-myristate 13-acetate (PMA) plus ionomycin or OVA_257–264_ in the presence of monensin before staining with a fixation/permeabilization kit (554774, BD Biosciences) according to the manufacturer’s instructions. For intracellular IL-12p40 detection, enriched splenic DCs were stimulated for 4 h with LPS or poly I:C in the presence of GolgiStop before staining using a fixation/permeabilization kit (554774, BD Biosciences) according to the manufacturer’s instructions. Transcription factor staining was performed with FOXP3/transcription factor staining buffer set (00-5523-00, eBioscience) according to the manufacturer’s instructions. Lysotracker staining was performed with LysoTracker Red DND-99 dye (L7528, Invitrogen) according to the manufacturer’s instructions. pHrodo Green Dextran (P35368, Invitrogen) staining was performed according to the manufacturer’s instructions. Flow cytometry data were acquired on LSRII, LSR Fortessa or Symphony A3 (BD Biosciences) using BD FACSDiva software (v8) and analysed using FlowJo software (Tree Star; v10). 7-Aminoactinomycin D (7AAD; A9400, 1:200, Sigma) or fixable viability dye (65-0865-14, 1:1,000, eBioscience) was used for dead-cell exclusion. The following fluorescent conjugate-labelled antibodies were used: PE-Cy7–anti-CD11c (N418, 60-0114, 1:200, Tonbo Biosciences); FITC–anti-FOXP3 (FJK-16s, 11-5773-82, 1:200), PE-Cyanine7–anti-T-bet (4B10, 25-5825-82, 1:100), APC-eFluor 780–anti-MHCII (M5/114.15.2, 47-5321-82, 1:400), PE-Cyanine7–anti-CD24 (M1/69, 25-0242-82, 1:400), FITC–anti-CD86 (GL1, 11-0862-82, 1:200), PE–anti-IL-12/IL-23 p40 (C17.8, 12-7123-82, 1:200), PE–anti-LAMP1 (eBioH4A3, 12-1079-42, 1:400) (all from eBioscience); Brilliant Violet 510–anti-CD4 (RM4-5, 100559, 1:200), AF700–anti-CD8α (53-6.7, 100730, 1:200), Brilliant Violet 785–anti-TCRβ (H57-597, 109249, 1:200), PE–anti-CD45.2 (104, 109808, 1:400), PE/Dazzle 594–anti-PD-1 (29F.1A12, 135228, 1:400), Alexa Fluor 647–anti-granzyme B (GB11, 515405, 1:100), PE-Cyanine7–anti-IFNγ (XMG1.2, 505826, 1:200), Brilliant Violet 421–anti-TNF (MP6-XT22, 506328, 1:200), APC–anti-IL-4 (11B11, 504106, 1:200), Pacific Blue–anti-IL-17A (TC11-18H10.1, 506918, 1:200), Brilliant Violet 711–anti-TIM-3 (RMT3-23, 119727, 1:400), Brilliant Violet 650–anti-CD44 (1M7, 103049, 1:400), PE-Cyanine7–anti-CD62L (MEL-14, 104417, 1:400), APC–anti-CD69 (H1.2F3, 104514, 1:200), Brilliant Violet 650–anti-CD11b (M1/70, 101259, 1:200), APC–anti-XCR1 (ZET, 148206, 1:400), APC–anti-CD103 (2E7, 121414, 1:400), Pacific Blue–anti-Ki67 (16A8, 652422, 1:400) (all from BioLegend); PE–anti-IL-2 (JES6-5H4, 554428, 1:200), Brilliant Violet 605–anti-Ly108 (13G3, 745250, 1:200) (from BD Biosciences); Alexa Fluor 647–anti-TCF1 (C63D9, 6709, 1:100, Cell Signaling Technology). Migratory cDC1s and cDC2s in tumour dLN were respectively gated as F4/80^−^Ly6C^−^CD11c^lo^MHCII^hi^CD103^+^CD11b^–^ and F4/80^−^Ly6C^−^CD11c^lo^MHCII^hi^CD103^–^CD11b^+^, as described^58^. Intratumoral cDC1s and cDC2s were respectively gated as CD45^+^F4/80^−^Ly6C^−^CD11c^+^MHCII^+^CD103^+^CD11b^–^ and CD45^+^F4/80^−^Ly6C^−^CD11c^+^MHCII^+^CD103^–^CD11b^+^, as described^19^.

### Antigen presentation assays

For in vitro assays, cDC1s and cCD2s were sorted from spleen, pulsed with 200 μg ml^−1^ OVA protein (Low Endo, LS003059, Worthington), 250 pg ml^−1^ OVA_257–264_ (vac-sin, InvivoGen) or 3 μg ml^−1^ OVA_323–339_ (vac-isq, InvivoGen) for 2 h, then washed twice and cultured with naive CD44^low^CD62L^high^ OT-I or OT-II cells for three days. For HKLM-OVA antigen cross-presentation assay, sorted cells were cocultured with 1 × 10^7^ HKLM-OVA and OT-I cells for 3 days as previously described^59^. [^3^H]Thymidine (PerkinElmer) was added to the culture 8 h before cells were collected to measure proliferation. Where indicated, cDC1s or cDC2s were incubated with OVA in RPMI 1640 medium lacking an individual amino acid or amino acid-free medium supplemented with an individual amino acid for 2 h, irradiated and then cocultured with T cells. For treatment with tumour cell culture supernatant, cDC1s or cDC2s were pulsed with OVA in the presence of MC38 or B16F10 culture supernatant or tumour cell culture supernatant supplemented with an individual amino acid for 2 h, followed by irradiation and coculture with OT-I or OT-II cells. For in vivo priming assay, 1 × 10^6^ CFSE-labelled naive CD45.1^+^ OT-I cells were transferred into mice intravenously, followed by intravenous injection with 20 μg OVA 24 h later. Three days after OVA immunization, spleens were collected, and the proliferation of OT-I cells was examined by CFSE dilution with flow cytometry.

### ELISA

Culture supernatant from in vitro antigen presentation assays was collected, and the levels of IL-2 and IFNγ were determined using IL-2 (88-7024-22, Thermo Fisher Scientific) and IFNγ (88-7314-22, Thermo Fisher Scientific) ELISA kits according to manufacturer’s instructions.

### DQ-ovalbumin degradation assay

Splenic cDC1s were sorted from wild-type and *Flcn*^*ΔDC*^ mice as described above. Sorted cells were incubated with 50 μg ml^−1^ DQ-Ovalbumin (DQ-OVA; D-12053, Thermo Fisher Scientific) for 0, 30, 60 or 120 min. DQ-OVA is a self-quenched OVA conjugate that emits green fluorescence upon hydrolysis by proteases. Cells were washed with PBS at the indicated times and analysed for DQ-OVA release as assessed positive FITC (FITC^+^) staining as described previously^59^.

### Tumour model and treatments

B16F10 cell line was purchased from ATCC. MC38, MC38-OVA and B16-OVA cell lines were provided by D. Vignali. B16-FLT3L cell line was provided by D. Green. B16F10 cell line expressing ZsGreen (B16-ZsGreen) was generated by lentiviral transduction of pHIV-ZsGreen construct (18121, Addgene) into B16F10 tumour cells, which were sorted based on expression of ZsGreen. These cell lines are not on the list of commonly misidentified cell lines (International Cell Line Authentication Committee). Cell lines used in this study were not independently authenticated or tested for mycoplasma contamination. All cell lines were maintained at 37 °C with 5% CO_2_ in DMEM supplemented with 10% (v/v) FBS and 1% (v/v) penicillin-streptomycin. Mice were injected subcutaneously with 5 × 10^5^ MC38, B16-OVA or B16-ZsGreen cells in the right flank. After tumour inoculation, mice were randomized and assigned to different groups for treatments. Glutamine was injected into tumours at a dose of 200 mg kg^−1^ per mouse daily starting from day 5 after tumour inoculation and for 10 consecutive days thereafter. Anti-PD-1 antibody (J43, Bio X Cell) or rat IgG2b isotype control (LTF-2, Bio X Cell) was injected intraperitoneally three times at a dose of 200 μg in 100 μl PBS at days 7, 10 and 13 after inoculation of MC38 cells. Anti-PD-L1 antibody (10F.9G2, Bio X Cell) or rat IgG2b isotype control (LTF-2, Bio X Cell) was injected intraperitoneally three times at a dose of 200 μg in 100 μl PBS at days 9, 12 and 15 after inoculation of B16-OVA cells. Mice with complete tumour rejections from intratumoral glutamine injection and anti-PD-1 combination therapy were rechallenged with 1 × 10^6^ MC38 cells after 60 days. Tumours were measured every two days with digital callipers and tumour volumes were calculated by the formula: length × width × (length × width)^0.5^ × π/6. Tumour size limits were approved to reach a maximum of 3,000 mm^3^ or ≤20% of body weight (whichever was lower) by the IACUC at St. Jude Children’s Research Hospital.

To analyse tumour antigen-specific immune responses, 1 × 10^6^ MC38-OVA cells were injected subcutaneously into mice. Tumour antigen-specific CD8^+^ T cells were analysed by H-2K^b^-OVA tetrameter staining for 30 min at room temperature. To prepare intratumoral lymphocytes, tumours were collected at day 15 after inoculation, excised, minced and digested with 1 mg ml^−1^ collagenase IV (Worthington) and 200 U ml^−1^ DNase I (Sigma) for 1 h at 37 °C. To analyse DC migration, tumour dLNs (including inguinal and axillary LN) were collected and digested with 1 mg ml^−1^ collagenase IV (Worthington) and 200 U ml^−1^ DNase I (Sigma) for 30 min at 37 °C.

### Generation of CRISPR–Cas9 knockout tumour cell lines

MC38 or B16-OVA cells were transduced with lentivirus of pLenti-Cas9-GFP (86145, Addgene). Cas9-expressing (GFP^+^) cells were sorted, and expression of Cas9 protein was confirmed by immunoblot analysis (data not shown). Cas9-expressing MC38 or B16-OVA cells were then transduced with lentivirus expressing Ametrine and control sgRNA (sgNTC: ATGACACTTACGGTACTCGT) or sgRNA targeting *Slc38a2* (sg*Slc38a2*: ATTAAATACTGACATTCCAA) as previously described^60^. After sorting of Ametrine^+^ cells, cells were expanded, and deletion of SLC38A2 was verified by immunoblot analysis. For tumour growth, 1 × 10^6^ sgNTC- or sg*Slc38a2*-transduced, Cas9-expressing MC38 or B16-OVA cells were injected subcutaneously into mice.

### DC transfer and adoptive T cell transfer for tumour therapy

For DC transfer experiments, freshly isolated splenic cDC1s were used following an established strategy^30^. In brief, B16-FLT3L cells (2.5 × 10^6^) were injected subcutaneously into both flanks of wild-type mice to expand cDC1s. Spleens were collected 10 days after tumour inoculation, and cDC1s were enriched using the CD8^+^ DC isolation kit (130-091-169, Miltenyi Biotec). Purified cDC1s were pulsed with 100 μg ml^−1^ OVA (low Endo, Worthington) together with 20 μg ml^−1^ poly I:C (InvivoGen) for 2 h in RPMI 1640 medium containing 10% dialysed FBS with or without glutamine. cDC1s were washed and transferred (1 × 10^6^ cells per mouse) subcutaneously adjacent to the tumours at day 5 after B16-OVA inoculation.

For OT-I cell transfer experiments, naive OT-I cells were isolated using a naive CD8α^+^ T cell isolation kit (130-096-543; Miltenyi Biotec) according to the manufacturer’s instructions. Purified naive OT-I cells were activated using 10 μg ml^−1^ anti-CD3 (2C11; Bio X Cell, BE0001-1) and 5 μg ml^−1^ anti-CD28 (37.51; Bio X Cell, BE0015-1) antibodies. Activated OT-I cells were then expanded in Click’s medium (Irvine Scientific) containing 10% dialysed FBS supplemented with or without glutamine in the presence of human recombinant IL-2 (20 IU ml^−1^; PeproTech), mouse IL-7 (12.5 ng ml^−1^; PeproTech) and IL-15 (25 ng ml^−1^; PeproTech) for 2–3 days before adoptive transfer. CFSE-labelled naive OT-I cells were transferred into PBS- or glutamine-supplemented MC38-OVA-bearing wild-type mice or MC38-OVA-bearing wild-type and *Slc38a2*^*ΔDC*^ or wild-type and *Flcn*^*ΔDC*^ mice on day 7 after tumour inoculation, followed by analysis of their proliferation (based on CFSE dilution) in the tumour dLNs on day 2 after adoptive transfer. Alternatively, activated OT-I cells were transferred into PBS- or glutamine-supplemented B16-OVA-bearing wild-type mice or B16-OVA-bearing wild-type and *Slc38a2*^*ΔDC*^ or wild-type and *Flcn*^*ΔDC*^ mice on day 12 after tumour inoculation, followed by their analysis in the tumour on day 7 after adoptive transfer as indicated in the figures and their legends. Where indicated, naive Cas9-expressing OT-I cells from Cas9 mice were activated and transduced with sgRNA targeting *Slc38a2* or control sgRNA as described above. Ametrine^+^ transduced cells were sorted before adoptive transfer into recipients.

### In vivo killing assay

In vivo killing assay was performed as previously described^24^. In brief, splenocytes were pulsed with OVA_257–264_ or PBS at 37 °C for 1 h. These antigen- or PBS-pulsed splenocytes were then labelled with CFSE or CellTrace Violet, respectively, at 37 °C for 15 min, then mixed at 1:1 ratio and a total of 1 × 10^7^ cells were transferred into MC38-OVA-bearing wild-type mice at day 12 that were treated with PBS or glutamine intratumorally daily starting at day 5 after tumour inoculation, followed by analysis of in vivo cytotoxicity against these splenocytes at 24 h after injection.

### Immunoprecipitation and immunoblot analysis

For FLCN immunoprecipitation in Fig. 3a, HEK293T cells were starved with glutamine-free medium for 0.5, 1, 2, 3 h or not starved; and in Fig. 3b, HEK293T cells were starved with glutamine-free medium for 3 h, followed by the addition of 2 mM glutamine for 10 or 15 min. The cells were then lysed in CHAPS buffer (0.3% CHAPS, 10 mM β-glycerol phosphate, 10 mM pyrophosphate, 40 mM HEPES pH 7.4, 2.5 mM MgCl_2_) supplemented with protease inhibitor cocktail (04693124001, Roche) for 30 min. The cell lysates were cleared by centrifugation and mixed with anti-HA magnetic beads (88837, Thermo Fisher Scientific) at 4 °C for 4 h. For immunoprecipitation of GATOR1 or GATOR2 complex, the cleared cell lysates were incubated with anti-DEPDC5 (for GATOR1) and anti-WDR24 (for GATOR2) antibodies and control IgG (3000-0-AP, ProteinTech) at 4 °C overnight, followed by a further incubation with protein A/G agarose beads (sc-2003, Santa Cruz) for 2 h. Immunoprecipitated complexes were washed three times with CHAPS buffer and subjected to immunoblot analysis. For immunoblot analysis, tumour cell lines (0.5 × 10^6^) or splenic cDC1s (0.1–0.15 × 10^6^) were collected and lysed in RIPA buffer (9806, Cell Signaling Technology), resolved in 4–12% Criterion XT Bis-Tris Protein Gel (Bio-Rad) and transferred to PVDF membrane (1620177, Bio-Rad). Membranes were blocked using 5% BSA for 1 h and then incubated with primary antibodies overnight (see below). After washing three times with TBST, the membranes were incubated with 1:5,000-diluted HRP-conjugated anti-mouse IgG (W4021, Promega) for 1 h. Following another three washes, the membranes were imaged by ODYSSEY Fc Imager (LI-COR). For immunoblot analysis of SLC38A2, cell lysates were treated with PNGase F (P0704S, New England Biolabs) to remove N-linked oligosaccharides according to the manufacturer’s instructions. The following antibodies were used: anti-β-Actin (3700), anti-GAPDH (D16H11), anti-Lamin B1 (D4Q4Z), anti-HA (3724), anti-MIOS (13557), anti-WDR59 (53385) (all were used at 1:1,000 dilution and from Cell Signaling Technology); anti-Cathepsin D (AF1029, R&D); anti-FLCN (ab124885), anti-DEPDC5 (ab213181), anti-SEH1L (ab218531) (all were used at 1:1,000 and from Abcam); anti-SEC13 (sc-514308); anti-NPRL2 (sc-376986) (both were used at 1:1,000 and from Santa Cruz); anti-Flag (F1804, 1:1,000, Sigma); anti-TFEB (A303-673A, 1:1,000, Bethyl Laboratories); anti-SLC38A2 (BMP081, 1:1,000, MBL); anti-WDR24 (20778-1-AP, 1:1,000, ProteinTech); and anti-NPRL3 (NBP-97766, 1:1,000, Novus Biologicals).

### Cytosolic and nuclear cell fractionation

For TFEB cytosolic and nuclear cell fractionation analysis in Fig. 4k and Extended Data Fig. 9d, freshly isolated splenic cDC1s (3 × 10^6^) from B16-FLT3L tumour-bearing mice were incubated in glutamine-sufficient medium or starved with glutamine-free medium for 3 h. The cells were washed twice with ice-cold PBS and collected into cytosol extraction buffer (150 mM NaCl; 50 mM HEPES, pH 7.4; and 0.025% (w/v) digitonin) supplemented with protease and phosphatase inhibitor cocktail (Roche). The samples were incubated on ice for 10 min, followed by centrifugation at 980 g for 5 min at 4 °C to pellet the nuclei, and the supernatant (cytoplasmic fraction) was further cleared by centrifugation at 13,000 g for 5 min. The nuclear pellet for each sample was washed three times with cytosol extraction buffer and lysed in RIPA buffer supplemented with protease and phosphatase inhibitor cocktail for 40 min on ice. After centrifugation at 14,000 g for 20 min, the resulting supernatant was used as the nuclear fraction.

### Immunofluorescence

Sort-purified cDC1s were allowed to adhere to poly-l-lysine-coated coverslips prior to fixation with 4% paraformaldehyde (PFA) for 10 min. Cells were then permeabilized with PBS containing 0.1% Triton-100 for 3 min prior to blocking with PBS containing 2% bovine serum albumin, 5% normal goat serum and 0.05% Tween-20. Cells were incubated overnight at 4 °C in blocking buffer containing anti-EEA1 antibody (3288, C45B10, 1:250; Cell Signaling Technology) followed by Alexa Fluor 488-conjugated anti-rabbit secondary antibody (A11008, 1 μg ml^−1^; Thermo Fisher Scientific) and Alexa Fluor 568-conjugated phalloidin to detect F-Actin (A12380, 1 U ml^−1^; Thermo Fisher Scientific). Coverslips were mounted in Vectashield Vibrance (Vector Labs), and images were acquired using a Marianas spinning disk confocal microscope (Intelligent Imaging Innovations) equipped with SoRa (Yokagawa), Prime 95B CMOS camera (Photometrics) and a 1.45 NA 100× oil objective. Images were acquired using Slidebook software (version 6.0.24; 3i) and analysed using Imaris software (version x64 9.5.1; Bitplane).

### Metabolomics and mass spectrometry for detection of glucose and amino acids

Plasma and TIF were collected as previously described^7^. In brief, subcutaneous tumour tissues were cut into pieces and then centrifuged through a 0.22-μm nylon filter (CLS8169, Corning). The flow-through was collected as TIF. The matched blood was collected from the orbital venous plexus, and plasma supernatant was collected by centrifugation. TIF and plasma were flash-frozen with liquid nitrogen and stored at –80 °C before analysis. Tumour cell culture supernatant was collected from medium cultured with MC38 or B16F10 cells in RPMI 1640 medium supplemented with 0.6 mM glutamine. 1 × 10^6^ sorted splenic cDC1s or cDC2s from wild-type or *Slc38a2*^*ΔDC*^ mice were collected. sgNTC- or sg*Slc38a2*-transduced, Cas9-expressing MC38 cells were cultured in DMEM supplemented with 10% (v/v) FBS and 1% (v/v) penicillin-streptomycin. Then, the cells were collected and washed once with ice-cold PBS, and the metabolites were extracted using 750 μl of methanol:acetonitrile:water (5:3:2, v/v/v) and the supernatant was dried by lyophilization. Aliquots of 20−50 μl from plasma and TIF were extracted with at least 15-fold excess volume of the methanol:acetonitrile:water solution, and the supernatant was then collected and dried by lyophilization. Dried extracts containing the hydrophilic metabolites were dissolved in 40 μl of water:acetonitrile (8:2, v/v) and 10 μl were used in the procedure to derivatize amino acids as described previously^61^ with some modifications. In brief, the samples were placed into glass autosampler vials and then 35 μl of sodium borate buffer (100 mM, pH 9.0) was added and mixed by pipetting. Next, 10 μl of the 6-aminoquinolyl-*N*-hydroxysuccinimidyl carbamate (AQC, 10 mM in acetonitrile)-derivatizing reagent (Cayman Chemical) was added. The vial was sealed, mixed by vortexing, and then incubated at 55 °C for 15 min. The vial was cooled to room temperature and then 1 μl was analysed by liquid chromatography with tandem mass spectrometry (LC–MS/MS). An ACQUITY Premier UPLC System (Waters Corp) was used for the LC separations, using non-linear gradient conditions as follows: 0−0.4 min 3% B; 0.4−8 min 3 to 96% B (using curve no. 8 of the inlet condition in MassLynx); 8−12 min 96% B; 12−12.5 min 96 to 3% B; 12.5−14 min 3% B. Mobile phase A was water supplemented with 0.15% acetic acid, and mobile phase B was acetonitrile with 0.15% acetic acid. The column used was an Accucore C30 (50 × 2.1 mm, 2.6 μm) (Thermo Fisher Scientific), operated at 50 °C. The flow rate was 300 μl min^−1^ and the injection volume used was 1 μl. All LC–MS/MS solvents and reagents were the highest purity available (water, acetonitrile, acetic acid, boric acid, sodium hydroxide) and were purchased from Thermo Fisher Scientific. A Xevo TQ-XS Triple Quadrupole Mass Spectrometry (TQ-XS) (Waters Corp) equipped with a multi-mode ESI/APCI/ESCi ion source was employed as detector. The TQ-XS was operated in the positive ion mode using the multiple reaction monitoring mass spectrometry method (MRM). The MRM conditions were set to a minimum of 15 points per peak, with automatic dwell time. The operating conditions of the source were: Capillary Voltage 3.8 kV, Cone Voltage 40 V, Desolvation Temp 550 °C, Desolvation Gas Flow 1,000 l h^−1^, Cone Gas Flow 150 l h^−1^, Nebuliser 7.0 Bar, Source Temp 150 °C. Authentic amino acids standards were purchased from Sigma-Aldrich and employed to establish the MRM conditions and calibration curves. The acquired MRM data were processed using the software application Skyline (version 21.2; MacCoss Lab Software).

### Quantification of amino acid uptake

Sorted splenic cDC1s and cDC2s or sgNTC- and sg*Slc38a2*-transduced Cas9-expressing MC38 cells were washed once with PBS and were then plated into 6-well plates at 1 × 10^6^ cells per well in RPMI 1640 medium containing 10% dialysed FBS and 2 mM [^13^C_5_]glutamine for 10 min. In certain experiments, cells were incubated with medium containing [^13^C]glutamine, [^13^C]alanine, [^13^C]serine, [^13^C]threonine, [^13^C]cysteine or [^13^C]asparagine (Cambridge Isotope Laboratories) for 10 min. The cells were subsequently washed once with ice-cold PBS, and the polar metabolites were extracted using 1 ml of methanol:acetonitrile:water (5:3:2, v/v/v) and the supernatant was dried by lyophilization. The dried extracts containing the hydrophilic metabolites were dissolved in 30 μl of water:acetonitrile (8:2, v/v) and 10 μl were used for the glutamine-derivatization procedure as described previously^61^ with minor modifications. In brief, the samples were placed into glass autosampler vials and then 35 μl of sodium borate buffer (100 mM, pH 9.0) was added, followed by mixing with pipetting. Next, 10 μl of the 6-aminoquinolyl-*N*-hydroxysuccinimidyl carbamate (AQC, 10 mM in acetonitrile)-derivatizing reagent (Cayman Chemical) was added. The vial was sealed, mixed by vortex, and incubated at 55 °C for 15 min. The vial was cooled to room temperature, and then 15 μl of the sample was analysed by LC–MS/MS. A Vanquish Horizon UHPLC (Thermo Fisher Scientific) was used for the LC separations, using non-linear gradient conditions as follows: 0−1 min 3% B; 1−22 min 3 to 96% B (using curve no. 8, Thermo Scientific SII for Xcalibur); 22−25 min 96% B; 25−26 min 96 to 3% B; 26−30 min 3% B. Mobile phase A was water supplemented with 0.15% acetic acid, and mobile phase B was acetonitrile with 0.15% acetic acid. The column used was an Accucore C30 (250 × 2.1 mm, 2.6 μm) (Thermo Fisher Scientific), operated at 50 °C. The flow rate was 300 μl min^−1^ and the injection volume used was 15 μl. All LC–MS/MS solvents and reagents were the highest purity available (water, acetonitrile, acetic acid, boric acid, sodium hydroxide) purchased from Thermo Fisher Scientific. A Q Exactive hybrid quadrupole-Orbitrap mass spectrometer (QE-MS) (Thermo Fisher Scientific) equipped with a HESI-II probe was employed as detector. The QE-MS was operated in the positive ion mode using targeted selected ions monitoring followed by a data-dependent MS/MS method (tSIM/dd-MS^2^). The QE-MS was operated at a resolution of 140,000 (FWHM, at 200 *m*/*z*), AGC targeted of 1 × 10^6^, max injection time 100 ms. For the dd-MS^2^ conditions a resolution of 35,000 was used, AGC targeted of 1 × 10^5^, maximum injection time 50 ms, loop count 8, MS^2^ isolation width 0.4 *m*/*z* and NCE 35. The operating conditions of the source were: Sheath gas flow 45; aux gas flow 8; sweep gas 1; spray voltage 3.8 kV in positive ion mode; capillary temperature 325 °C; S-lenses RF level 55; aux gas heater at 325 °C. Authentic unlabelled and [^13^C_5_]glutamine standards were purchased from Sigma-Aldrich. The relative contents of intracellular glutamine [M+0] and [^13^C_5_]glutamine [M+5] were determined from the tSIM/dd-MS^2^ data as the corresponding parent/daughter ions 317.1230/171.0554 for [M+0] and 322.1663/171.0554 for [M+5]. The data was processed using the Xcalibu software (Thermo Fisher Scientific).

### RNA isolation and gene expression profiling

RNA was isolated and purified from various cell types using the RNeasy Micro Kit (74004, Qiagen) according to the manufacturer’s instructions. cDNA synthesis was performed using the High Capacity cDNA Reverse Transcription Kit (4368813, Thermo Fisher Scientific) according to the manufacturer’s instructions. Real-time PCR was performed on the QuantStudio 7 Flex System (Applied Biosystems) using the PowerSYBR Green PCR Master Mix (4367659, Thermo Fisher Scientific). The sequences for mouse *Flcn* primers were previously described^55^. The primers for detection of glutamine transporters are listed below: *Slc1a5*-F: CATCAACGACTCTGTTGTAGACC, *Slc1a5*-R: CGCTGGATACAGGATTGCGG; *Slc6a14*-F: GACAGCTTCATCCGAGAACTTC, *Slc6a14*-R: ATTGCCCAATCCCACTGCAT; *Slc6a19*-F: CAGGTGCTCAGGTCTTCTACT, *Slc6a19*-R: CGATCACAGAATCCATCTCACAA; *Slc7a5*-F: ATATCACGCTGCTCAACGGTG, *Slc7a5*-R: CTCCAGCATGTAGGCGTAGTC; *Slc7a6*-F: GCCTGCGTATGTCTGCTGA, *Slc7a6*-R: GCCCATGATAATGATGGCAATGA; *Slc7a7*-F: CACCACCAAGTATGAAGTGGC, *Slc7a7*-R: CCCTTAGGGGAGACAAAGATGC; *Slc7a8*-F: TGTGACTGAGGAACTTGTGGA, *Slc7a8*-R: GTGGACAGGGCAACAGAAATG; *Slc7a9*-F: GAGGAGACGGAGAGAGGATGA, *Slc7a9*-R: CCCCACGGATTCTGTGTTG; *Slc38a1*-F: AGCAACGACTCTAATGACTTCAC, *Slc38a1*-R: CCTCCTACTCTCCCGATCTGA; *Slc38a2*-F: TAATCTGAGCAATGCGATTGTGG, *Slc38a2*-R: AGATGGACGGAGTATAGCGAAAA; *Slc38a3*-F: GGAGGGGCTTCTACCAGTG, *Slc38a3*-R: GGAAAAGGATGATGCCCGTATTG; *Slc38a4*-F: GCGGGGACAGTATTCAGGAC, *Slc38a4*-R: GGAACTTCTGACTTTCGGCAT; *Slc38a5*-F: CTACAGGCAGGAACGCGAAG, *Slc38a5*-R: GGTTGAACACTGACATTCCGA; *Actb*-F: GGCACCACACCTTCTACAAT, *Actb*-R: CTTTGATGTCACGCACGATTTC. For microarray analysis, splenic cDC1s were sort-purified from wild-type (*n* = 2) and *Flcn*^*ΔDC*^ mice (*n* = 3) as described above. RNA was extracted and purified, and 125 ng RNA was used to profile with Affymetrix Mouse Clariom S Assay. For microarray analysis, the gene expression probe signals were quantile-normalized and summarized by the RMA algorithm by Affymetrix Expression Console (version 1.4.1), then the differential gene expression analysis was performed by R package limma (version 3.46.0). False discovery rate (FDR) was estimated by Benjamini–Hochberg method. Heat maps were generated using ComplexHeatmap (version 2.6.2) to show the average expression of genes from biological replicates of the same genotype. Microarray data have been deposited into the GEO series database under accession GSE210155.

### ATAC-seq and data analysis

#### Library preparation

The ATAC-seq library was prepared as previously described^60^. In brief, splenic cDC1s from wild-type and *Flcn*^*ΔDC*^ mice (*n* = 4 per genotype) were isolated as described above. A total of 5 × 10^4^ cells for each sample were used for the ATAC-seq library construction. After lysing in 50 μl ATAC-seq lysis buffer (10 mM Tris-HCl, pH 7.4, 10 mM NaCl, 3 mM MgCl_2_, 0.1% IGEPAL CA-630) on ice for 10 min, the resulting nuclei pellet was resuspended in 50 μl transposase reaction mix (25 μl 2 × TD buffer, 22.5 μl nuclease-free water, and 2.5 μl transposase) and incubated for 30 min at 37 °C. The tagged DNA was cleaned up using the Qiagen MinElute kit (Qiagen). A first round PCR with 5 cycles was performed to amplify and barcode the tagged DNA. The optimal cycle of further amplification was determined by real-time PCR (KAPA SYBRFast system; Kapa Biosystems). The final PCR products were purified using AMPure XP beads (Beckman Coulter). The fragment distribution of each library was checked by a TapeStation System (Agilent Technologies) and then sequenced on an Illumina NovaSeq with ~300 million reads per sample.

#### Data analysis

ATAC-seq analysis was performed as described previously^60^. In brief, the paired-end fastq files obtained from NovaSeq were trimmed for Nextera adapter by trimmomatic (version 0.36, paired-end mode, with parameter LEADING:10 TRAILING:10 SLIDINGWINDOW:4:18 MINLEN:25). BWA (version 0.7.16) was used to align reads to mouse genome mm10 with default parameters. Resulting BAM files were filtered to remove duplicated reads (marked by Picard (version 2.9.4)) and to remove mitochondrial reads. After adjustment of Tn5 shift (reads were offset by +4 bp for the sense strand and −5 bp for the antisense strand), the reads were separated into nucleosome-free, mononucleosome, dinucleosome and trinucleosome by fragment size. All samples in this study had approximately 1 × 10^7^ nucleosome-free reads, indicative of good data quality. Next, these nucleosome-free reads were used for peak calling by MACS2 (version 2.1.1.20160309, with default parameters with ‘–extsize 200–nomodel’) with a higher cut-off (MACS2 −q 0.05). The consensus peaks for each group were further generated by keeping peaks that were presented in at least 50% of the replicates. The reproducible peaks were merged between wild-type and FLCN-deficient cDC1s if they overlapped by 100-bp and then were counted from each of the 8 samples by bedtools (version 2.25.0). Transcription factor footprinting activity were inferred and visualized using the RGT HINT software (version 0.13.2)^62^. Raw and processed ATAC-seq data have been deposited into the GEO series database under accession GSE210155.

### scRNA-seq and data analysis

#### Library preparation

For scRNA-seq analysis in Fig. 1 and Extended Data Fig. 1, wild-type mice were challenged with MC38 colon adenocarcinoma cells, and treated with PBS or glutamine daily starting from day 5. DCs (CD45^+^CD64^−^Ly6C^−^CD11c^+^MHCII^+^), CD45^+^ non-macrophage immune cells (CD45^+^CD64^−^), macrophages (CD45^+^CD64^+^), and CD45^−^ tumour and other non-immune cells in the tumour tissues were sorted at 15 d after tumour challenge and mixed at a 5:4:1:1 ratio (to ensure that sufficient numbers of the less abundant DCs and non-macrophage immune cells were profiled; *n* = 2 biological replicates per group). For scRNA-seq analysis in Extended Data Figs. 7 and 8, wild-type and *Flcn*^*ΔDC*^ mice (*n* = 2 per genotype) were challenged with MC38 cells. CD45^+^ cells and DCs (CD45^+^CD64^−^Ly6C^−^CD11c^+^MHCII^+^) in the tumour tissues were sorted at 15 days after tumour challenge and mixed at a 2:1 ratio. The cell mixture was centrifuged at 2,000 rpm for 5 min and then resuspended in 1× PBS (Thermo Fisher Scientific) plus 0.04% BSA (Amresco) with a final concentration of 1 × 10^6^ cells per ml. The single-cell suspensions were loaded onto a Chromium Controller and encapsulated into droplets. Chromium Next GEM Single Cell 3′ (version 3.1; for scRNA-seq analysis in Fig. 1 and Extended Data Fig. 1) or Next GEM Single Cell 5′ (version 2; for scRNA-seq analysis in Extended Data Figs. 7 and 8) and Gel Bead Kit (10x Genomics) were used for the library preparation following manufacture’s instruction. The final libraries were quality-checked by 2100 Bioanalyzer (Agilent Technologies) and quantified by Qubit Fluorometer (Invitrogen). The resulting libraries were sequenced on NovaSeq (Illumina) with paired-end reads of 26 (for Chromium Next GEM Single Cell 5′ kit) or 28 (for Chromium Next GEM Single Cell 3′ kit) cycles (for read 1, 90 cycles for read 2 and 10 cycles for index 1 and 2 separately). An average of 500 million reads per sample were obtained.

#### Data preprocessing and quality control

After raw sequencing data were de-multiplexed by bcl2fastq (version 2.20.0.422), the Cell Ranger Single-Cell software suite (version 6.0; 10x Genomics) was used to process with the scRNA-seq FASTQ files. In brief, the FASTQ files were aligned to the mm10 mouse reference genome (ENSEMBL GRCm38). Gene expression was quantified by reads confidently mapped to the genome and assigned to cells by the cell barcodes. The output from Cell Ranger was imported into R (version 4.0.5) and analysed with Seurat (version 4.0.2). Cells with fewer than 200 genes detected, or with low unique molecular identifiers (UMI) counts (potentially dead cells) or unusually high UMI counts (potentially two or more cells in a single droplet) were removed. Cells with high percent (>5%) of reads mapping to mitochondrial genes (potentially dead cells) were also removed. Genes detected in fewer than three cells were discarded.

#### Clustering and cluster annotation

For unsupervised clustering and visualization, we normalized the expression level of each gene using NormalizeData with scale.factor as 1 × 10^6^ built in Seurat pipeline as described previously^60^. In brief, principal component analysis (PCA) was performed using the top 2,000 highly variable genes. The top 30 principal components were used to build a shared nearest neighbour (SNN) graph, and cells were clustered using the Louvain algorithm as implemented in a FindClusters function from the Seurat package with resolution as 0.5. The cluster-specific differentially expressed genes were calculated by FindAllMarkers function from Seurat. For the CD8^+^ T cell subset analysis, we subsetted the CD8^+^ T cells by gating on the high expression of the CD3 subunit genes (*Cd3e* or *Cd3d*) and *Cd8b* gene and performed unsupervised clustering using the same graph-based clustering method. The CD8^+^ T cell subsets were further characterized by the high expression of *Tcf7* (encodes TCF1) or *Havcr2* (encodes TIM-3). For DCs, we first subsetted cDCs with high expression of *Ptprc* and *Flt3*. A second-round of dimensionality reduction and unsupervised clustering were then performed. The cDC1 cell cluster was characterized by expression of *Clec9a* and *Xcr1*. cDC2 cell cluster was characterized by expression of *Cd209a*. cDC1 and cDC2 gene signatures were then generated by identifying the top and bottom 200 genes (ranking log_2_ fold change) of differential expression between cDC1s versus cDC2s. DCs that expressed *Ccr7*^63,64^ were further subclustered into cDC1-derived and cDC2-derived cells based on cDC1 and cDC2 gene signatures. The cDC1-derived *Ccr7*^+^ DC cluster was then merged with the cDC1 cluster, while cDC2-derived *Ccr7*^+^ DC cluster was then merged with the cDC2 cluster for subsequent differential expression analysis, which was performed by FindMarkers function from Seurat package. The activity scores of gene signatures were calculated by AddModuleScore function. The difference in gene signature activity was examined by non-parametric, two-tailed Wilcoxon rank sum test and visualized using violin plots. Raw and processed scRNA-seq data have been deposited into the GEO series database under accession GSE210155.

### Gene set enrichment analysis and signature curation

Genes were ranked by the fold change generated by the differential expression analysis. The pre-ranked gene set enrichment analysis (GSEA) was performed as previously described^65^ against gene sets from KEGG, BIOCARTA, PID, REACTOME, C7 immunological, GO and HALLMARK collections from the Molecular Signatures Database (mSigDB) (https://www.broadinstitute.org/gsea/msigdb/, version 7.4) and signatures curated from published papers, as follows. For CD8^+^ T cells, gene signatures of ‘early activation’ and ‘effector/cytokine’ were curated by a previous publication^24^. For cDC1s, the ‘MHCI antigen presentation’ signature and ‘DC activation’ signature were described in previous publications^24,64^. The set of ‘putative TFEB target genes’ signature was derived from a public dataset, which identified TFEB targets by integrating TFEB ChIP-seq analysis and TFEB overexpression^66^.

### Public bioinformatics dataset analysis

To examine the expression of glutamine transporters in tumour cells, DCs, macrophages, B cells, natural killer cells, CD8^+^ and CD4^+^ T cells and other immune cells from the TME, a human melanoma dataset^35^ (GSE72056) and a mouse tumour scRNA-seq dataset^34^ (GSE121861, profiling B16F10 melanoma, EMT6 breast mammary carcinoma, LL2 Lewis lung carcinoma, CT26 and MC38 colon carcinoma and Sa1N fibrosarcoma) were analysed with Seurat (version 4.0.2). Tumour cells and CD45^+^ immune cells from different mouse tumour models were pooled for analysis in GSE121861. Expression of glutamine transporters in the indicated cell types was visualized by DotPlot function. To compare the expression of *Slc1a5*, *Slc6a14*, *Slc6a19*, *Slc7a5*, *Slc7a6*, *Slc7a7*, *Slc7a8*, *Slc7a9*, *Slc38a1*, *Slc38a2*, *Slc38a3*, *Slc38a5*, *Slc38a7* and *Slc38a8* in different immune cell types, the Immgen Microarray Gene Skyline data^67^ was downloaded and visualized by the heatmap function in ComplexHeatmap R package.

Public microarray datasets profiling glutamine transporters in different immune cell subsets are available from the Immgen database (https://www.immgen.org/). KEGG, BIOCARTA, PID, REACTOME, C7 immunological, GO and HALLMARK collections were from the Molecular Signatures Database (mSigDB) (https://www.broadinstitute.org/gsea/msigdb/).

### Statistical analysis for biological experiments

Analyses of biological experiments (non-omics) were performed using Prism software (version 8; GraphPad) by two-tailed paired Student’s *t*-test, two-tailed unpaired Student’s *t*-test, or one-way ANOVA with Newman–Keuls’s test. Two-way ANOVA was performed for comparing tumour growth curves. The Mantel–Cox test was used for comparing mouse survival curves. Two-tailed Wilcoxon rank sum test was applied for differential expression or activity score analysis of scRNA-seq data. Two-tailed Kolmogorov–Smirnov test by GSEA was used for pathway activity score analysis of scRNA-seq data. Two-tailed unpaired Student’s *t*-test was used for transcription factor footprinting analysis of ATAC-seq peaks. *P* < 0.05 was considered significant, and the exact *P* values are provided in the source data that accompanies this manuscript. Data are presented as mean ± s.e.m. No statistical method was used to pre-determine the sample sizes, but our sample sizes are similar to those reported in other publications^16,17^. Age- and sex-matched mice with pre-determined genotypes were randomly assigned to control and experimental groups. No other randomization was performed. Data collection and analysis were not performed blind to the conditions of the experiments.

### Reporting summary

Further information on research design is available in the Nature Portfolio Reporting Summary linked to this article.

## Online content

Any methods, additional references, Nature Portfolio reporting summaries, source data, extended data, supplementary information, acknowledgements, peer review information; details of author contributions and competing interests; and statements of data and code availability are available at 10.1038/s41586-023-06299-8.

## Supplementary information

Supplementary Figures This file contains Supplementary Figs. 1 and 2. Supplementary Fig. 1: Gating strategies for flow cytometry analysis and cell sorting. **a**, Gating strategy for analysis of intratumoral immune cells from MC38 tumours. **b**, Gating strategy for analysis of adoptively transferred naive OT-I cell proliferation (based on CFSE dilution) in spleen after OVA immunization (in vivo priming assay). **c**, Gating strategy for analysis of adoptively transferred naive OT-I cell proliferation (based on CFSE dilution) in tumour-draining lymph nodes (dLNs) from MC38-OVA tumour-bearing mice. **d**, Gating strategy for analysis of migratory DCs in the dLNs from B16-ZsGreen tumour model (upper). Lower, B16F10 tumour cells serve as a negative control for gating ZsGreen^+^ cells. **e**, Gating strategy for analysis of adoptively transferred effector-like and stem-like populations among activated OT-I cells in B16-OVA tumours. Blue labels indicate the final populations of interest in the analysis. Supplementary Fig. 2: Uncropped immunoblot images with size marker indications.

Reporting Summary

Supplementary Table 1 List of substrate specificities, direction of transport and tissue expression of reported glutamine transporters. This file contains the literature-curated information regarding the substrate specificity, direction of transport and tissue expression of the individual glutamine transporters indicated.

Supplementary Table 2 Gene set enrichment analysis of KEGG, HALLMARK, and putative TFEB gene signatures in transcriptomes of FLCN-deficient versus wild-type cDC1s. This file contains the complete lists of enriched or downregulated (based on FDR < 0.25) signatures from gene set enrichment analysis (GSEA) of transcriptomes from FLCN-deficient versus wild-type cDC1s (**a**–**d**). The individual tabs show upregulated (**a**, **c**) or downregulated (**b**, **d**) signatures from KEGG (**a**, **b**) or HALLMARK combined with putative TFEB gene targets (**c**, **d**). Signatures are ranked by NES (normalized enrichment score). FDR q-val., false discovery rate q-value. KEGG and HALLMARK signatures are from the Molecular Signatures Database and the TFEB gene targets are curated from a published paper (see Methods).

## Data Availability

The authors declare that the data supporting the findings of this study are available within the paper and its Supplementary Information. All microarray, ATAC-seq and scRNA-seq data described in the manuscript have been deposited in the NCBI Gene Expression Omnibus (GEO) database and are accessible through GEO SuperSeries accession number GSE210155.  Source data are provided with this paper.

## Source data

Source Data Fig. 1
Source Data Fig. 2
Source Data Fig. 3
Source Data Fig. 4
Source Data Extended Data Fig. 1
Source Data Extended Data Fig. 2
Source Data Extended Data Fig. 3
Source Data Extended Data Fig. 4
Source Data Extended Data Fig. 5
Source Data Extended Data Fig. 6
Source Data Extended Data Fig. 7
Source Data Extended Data Fig. 8
Source Data Extended Data Fig. 9
Source Data Extended Data Fig. 10
