# Supplementary material for: SLC38A2 and glutamine signalling in cDC1s dictate anti-tumour immunity
Source: Nature. 2023 Jul 5;620(7972):200–8. doi: 10.1038/s41586-023-06299-8 (PMC10396969; doi:10.1038/s41586-023-06299-8)

nature portfolio

https://doi.org/10.1038/s41586-023-06299-8

Supplementary information

SLC38A2 and glutamine signalling in cDC1s dictate anti-tumour immunity

In the format provided by the authors and unedited

Supplementary Figure 1. Gating strategies for flow cytometry analysis and cell sorting

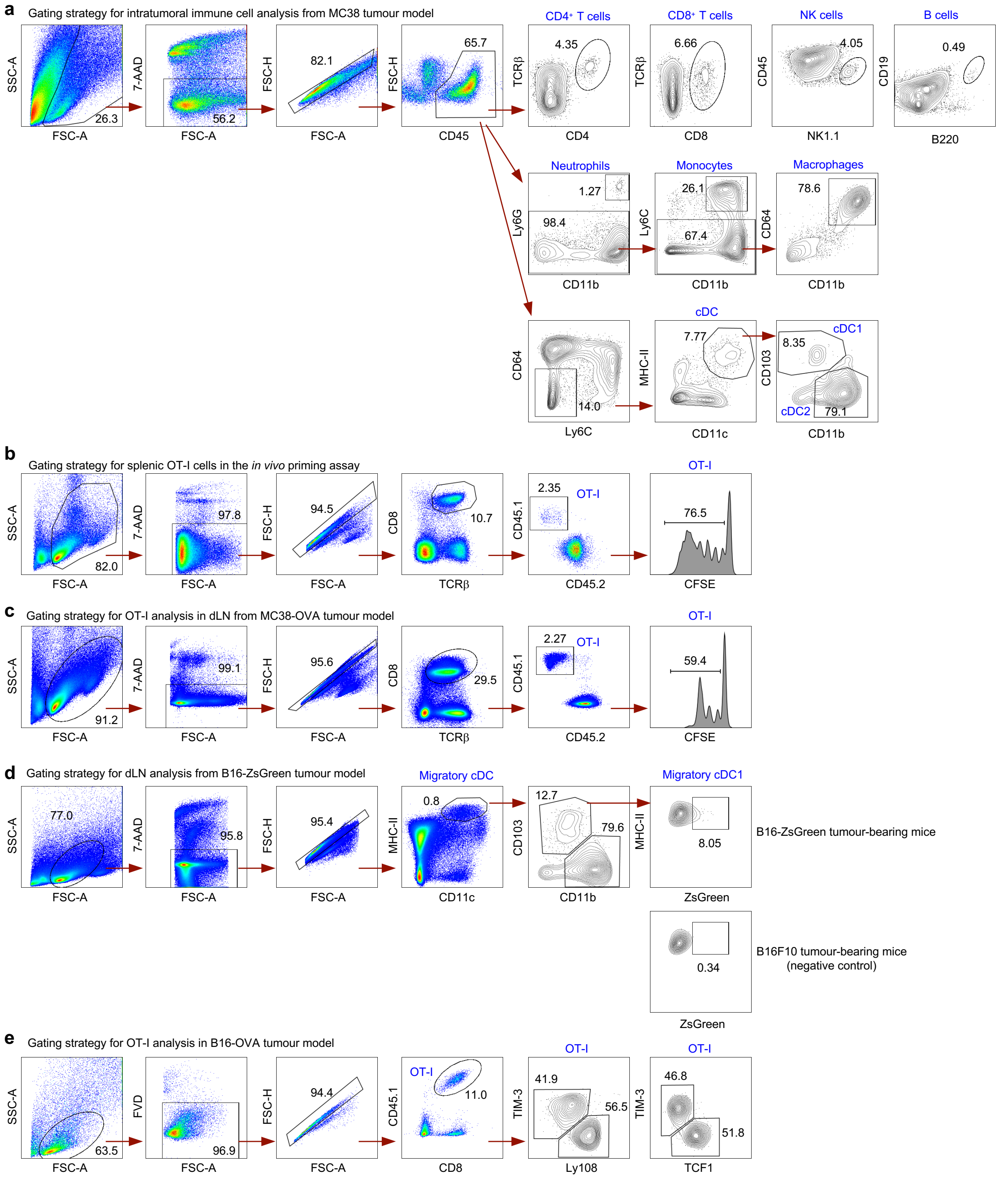

Supplementary Figure 2. Uncropped immunoblot images with size marker indications

Fig. 2g

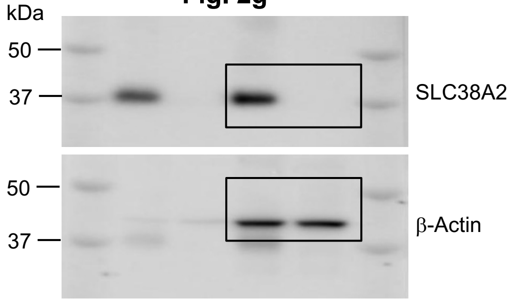

Fig. 3a

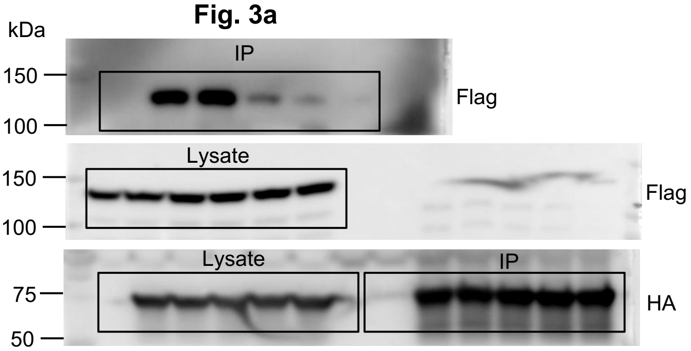

Fig. 3b

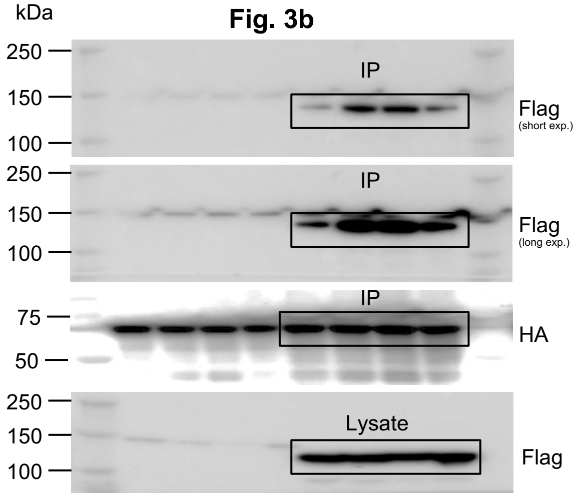

Fig. 4e

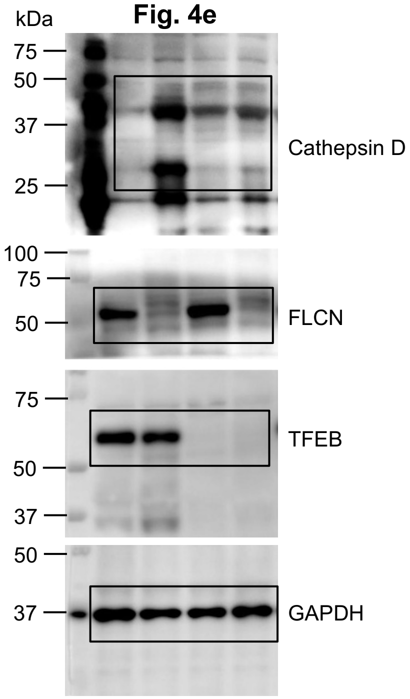

Fig. 4k

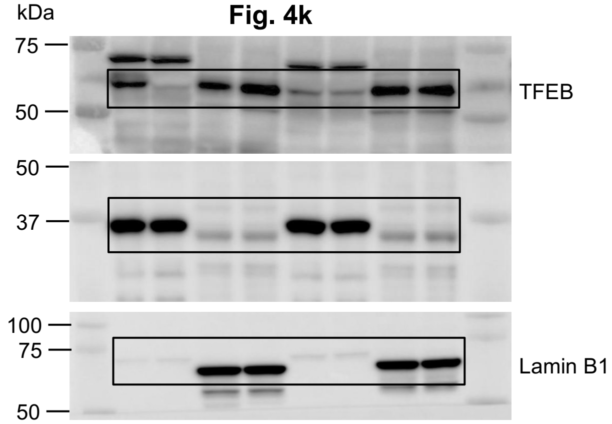

Extended Data Fig. 4d

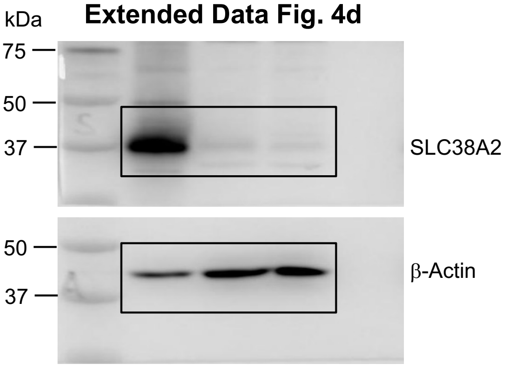

Extended Data Fig. 4g

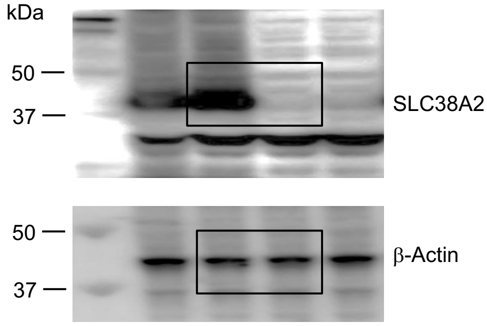

Extended Data Fig. 7a

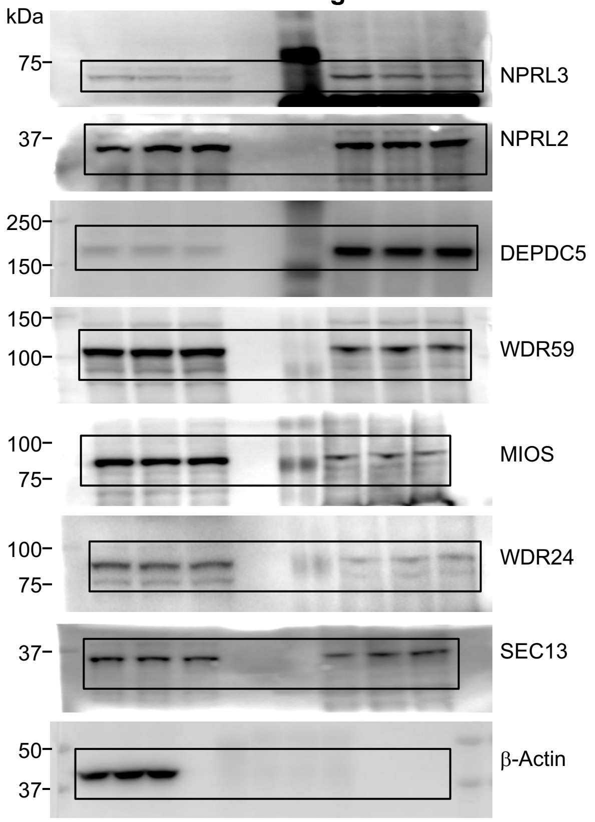

Extended Data Fig. 7b

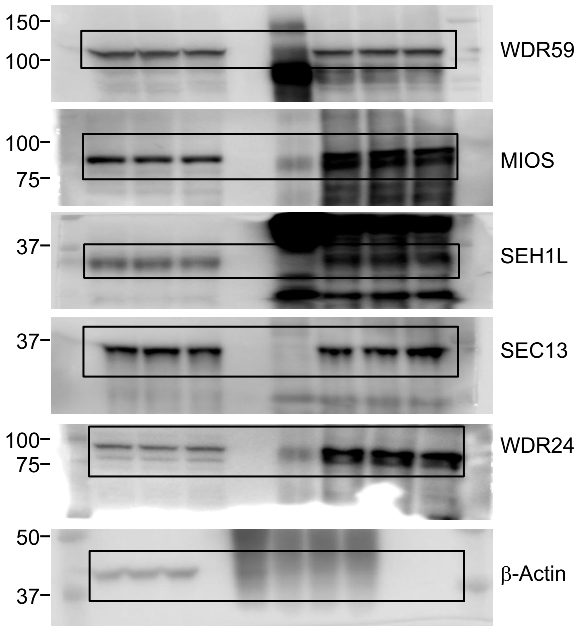

Extended Data Fig. 9d

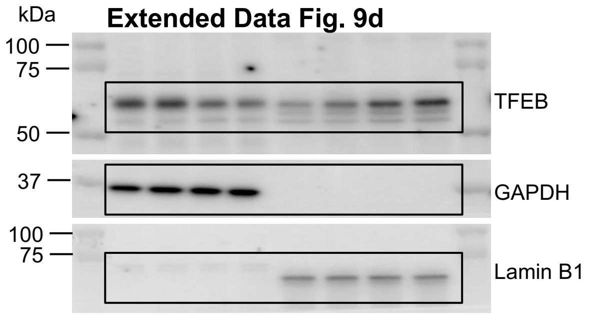

Extended Data Fig. 9i

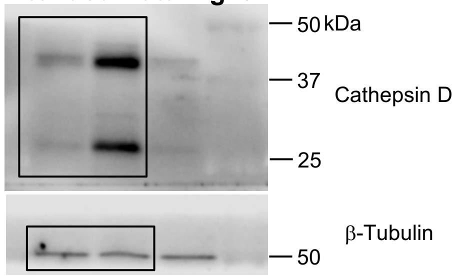

Supplement: Supplementary file 1 — This file contains Supplementary Figs. 1 and 2. Supplementary Fig. 1: Gating strategies for flow cytometry analysis and cell sorting. a, Gating strategy for analysis of intratumoral immune cells from MC38 tumours. b, Gating strategy for analysis of adoptively transferred naive OT-I cell proliferation (based on CFSE dilution) in spleen after OVA immunization (in vivo priming assay). c, Gating strategy for analysis of adoptively transferred naive OT-I cell proliferation (based on CFSE dilution) in tumour-draining lymph nodes (dLNs) from MC38-OVA tumour-bearing mice. d, Gating strategy for analysis of migratory DCs in the dLNs from B16-ZsGreen tumour model (upper). Lower, B16F10 tumour cells serve as a negative control for gating ZsGreen+ cells. e, Gating strategy for analysis of adoptively transferred effector-like and stem-like populations among activated OT-I cells in B16-OVA tumours. Blue labels indicate the final populations of interest in the analysis. Supplementary Fig. 2: Uncropped immunoblot images with size marker indications. [file 41586_2023_6299_MOESM1_ESM.pdf]
