# Supplementary material for: SLC38A2 and glutamine signalling in cDC1s dictate anti-tumour immunity
Source: Nature. 2023 Jul 5;620(7972):200–8. doi: 10.1038/s41586-023-06299-8 (PMC10396969; doi:10.1038/s41586-023-06299-8)
Supplement: Supplementary file 2 — Reporting Summary [file 41586_2023_6299_MOESM2_ESM.pdf]

nature portfolio

Corresponding author(s): Hongbo Chi

Last updated by author(s): May 24, 2023

Reporting Summary

Nature Portfolio wishes to improve the reproducibility of the work that we publish. This form provides structure for consistency and transparency in reporting. For further information on Nature Portfolio policies, see our Editorial Policies and the Editorial Policy Checklist.

Statistics

For all statistical analyses, confirm that the following items are present in the figure legend, table legend, main text, or Methods section.

n/a	Confirmed	
☐	☒	The exact sample size (*n*) for each experimental group/condition, given as a discrete number and unit of measurement
☐	☒	A statement on whether measurements were taken from distinct samples or whether the same sample was measured repeatedly
☐	☒	The statistical test(s) used AND whether they are one- or two-sided<br>*Only common tests should be described solely by name; describe more complex techniques in the Methods section.*
☐	☒	A description of all covariates tested
☐	☒	A description of any assumptions or corrections, such as tests of normality and adjustment for multiple comparisons
☐	☒	A full description of the statistical parameters including central tendency (e.g. means) or other basic estimates (e.g. regression coefficient) AND variation (e.g. standard deviation) or associated estimates of uncertainty (e.g. confidence intervals)
☐	☒	For null hypothesis testing, the test statistic (e.g. *F*, *t*, *r*) with confidence intervals, effect sizes, degrees of freedom and *P* value noted<br>*Give P values as exact values whenever suitable.*
☒	☐	For Bayesian analysis, information on the choice of priors and Markov chain Monte Carlo settings
☒	☐	For hierarchical and complex designs, identification of the appropriate level for tests and full reporting of outcomes
☒	☐	Estimates of effect sizes (e.g. Cohen's *d*, Pearson's *r*), indicating how they were calculated

Our web collection on statistics for biologists contains articles on many of the points above.

Software and code

Policy information about availability of computer code

Data collection	BD FACSDIva software (v8) was used to collect flow cytometry data on LSRII, Fortessa or Symphony A3 cytometers (BD Biosciences). Imaging SlideBook software (v6.0.24) was used to collect imaging data.
Data analysis	Flow cytometry analysis: FlowJo v10 (TreeStar) for flow cytometry results;<br>Statistics: GraphPad Prism v8 for statistics;<br>For microarray and sequencing analyses: R (v4.0.5)<br>Microarray analysis: Affymetrix Expression console (v1.4.1), R package limma (v3.46.0), ComplexHeatmap (v2.6.2).<br>ATAC-seq analysis: BWA (v0.7.16), MACS2 (v2.1.120160309), bedtools (v2.25.0), RGT HINT (v0.13.2).<br>scRNA-seq analysis; Cell Ranger (v6.0), Seurat R package (v4.0.2) with functions FindClusters, FindMarkers, and AddModuleScore, UMAP (Uniform Manifold Approximation and Projection), GSEA command-line software (v4.0.3), MSigDB (v7.3).<br>Imaging analysis: Imaris software (vx64 9.5.1).<br>Mass spectrometry analysis: Skyline (v21.2).

For manuscripts utilizing custom algorithms or software that are central to the research but not yet described in published literature, software must be made available to editors and reviewers. We strongly encourage code deposition in a community repository (e.g. GitHub). See the Nature Portfolio guidelines for submitting code & software for further information.

Data

Policy information about availability of data

All manuscripts must include a data availability statement. This statement should provide the following information, where applicable:

- Accession codes, unique identifiers, or web links for publicly available datasets
- A description of any restrictions on data availability
- For clinical datasets or third party data, please ensure that the statement adheres to our policy

The authors declare that the data supporting the findings of this study are available within the paper and its Supplementary Information. All microarray, ATAC-seq and scRNA-seq data described in the manuscript have been deposited in the NCBI Gene Expression Omnibus (GEO) database and are accessible through the GEO SuperSeries access number GSE210155 (https://www.ncbi.nlm.nih.gov/geo/query/acc.cgi?acc=GSE210155). Public scRNA-seq datasets are available through GSE72056 (https://www.ncbi.nlm.nih.gov/geo/query/acc.cgi?acc=GSE72056) and GSE121861 (https://www.ncbi.nlm.nih.gov/geo/query/acc.cgi?acc=GSE121861). Public microarray dataset profiling glutamine transporters in different immune cell subsets is available from the Immgen database (https://www.immgen.org/). KEGG, BIOCARTA, PID, REACTOME, C7 immunological, GO and HALLMARK collections were from the Molecular Signatures Database (mSigDB) (https://www.broadinstitute.org/gsea/msigdb/).

Human research participants

Policy information about studies involving human research participants and Sex and Gender in Research.

Reporting on sex and gender	N/A
Population characteristics	N/A
Recruitment	N/A
Ethics oversight	N/A

Note that full information on the approval of the study protocol must also be provided in the manuscript.

Field-specific reporting

Please select the one below that is the best fit for your research. If you are not sure, read the appropriate sections before making your selection.

☒ Life sciences ☐ Behavioural & social sciences ☐ Ecological, evolutionary & environmental sciences

For a reference copy of the document with all sections, see nature.com/documents/nr-reporting-summary-flat.pdf

Life sciences study design

All studies must disclose on these points even when the disclosure is negative.

Sample size	Sample sizes were selected based on those used in previous publications (Du et al. Nature 2018; Wang et al. Cell Res 2019).
Data exclusions	No data were excluded.
Replication	All the experimental findings were reliably reproduced as validated by at least three biological replicates in at least two independent experiments.
Randomization	Age- and sex-matched mice were assigned randomly to experimental and control groups.
Blinding	The investigators were not blinded to group allocation during data collection or analysis. This approach is considered standard for experiments of the type performed in this study, as the genetic background of mice must be predetermined prior to analysis.

Reporting for specific materials, systems and methods

We require information from authors about some types of materials, experimental systems and methods used in many studies. Here, indicate whether each material, system or method listed is relevant to your study. If you are not sure if a list item applies to your research, read the appropriate section before selecting a response.

Materials & experimental systems

n/a	Involved in the study
☐	☒ Antibodies
☐	☒ Eukaryotic cell lines
☒	☐ Palaeontology and archaeology
☐	☒ Animals and other organisms
☒	☐ Clinical data
☒	☐ Dual use research of concern

Methods

n/a	Involved in the study
☒	☐ ChIP-seq
☐	☒ Flow cytometry
☒	☐ MRI-based neuroimaging

Antibodies

Antibodies used

1. The following antibodies were used for cell culture: anti-CD3 (2C11; Bio X Cell, BE0002) and anti-CD28 (37.51; Bio X Cell, BE0015-1).

2. The following antibodies were used for in vivo treatments: anti-PD-1 antibody (J43, Bio X Cell), anti-PD-L1 antibody (10F.9G2, Bio X Cell) and rat IgG2b isotype control (LTF-2, Bio X Cell).

3. For flow cytometry analysis: 7-AAD (A9400, 1:200, Sigma) or fixable viability dye (65-0865-14; 1:1000, eBioscience) was used for dead-cell exclusion. The following fluorescent conjugate-labeled antibodies were used: PE-Cy7–anti-CD11c (N418, 60-0114, 1:200, Tonbo Biosciences); FITC–anti-FOXP3 (FJK-16s, 11-5773-82, 1:200), PE-Cyanine7–anti-T-bet (4B10, 25-5825-82, 1:100), APC-eFluor 780–anti-MHC-II (M5/114.15.2, 47-5321-82, 1:400), PE-Cyanine7–anti-CD24 (M1/69, 25-0242-82, 1:400), FITC–anti-CD86 (GL1, 11-0862-82, 1:200), PE–anti-IL-12/IL-23 p40 (C17.8, 12-7123-82, 1:200), PE–anti-LAMP1 (eBioH4A3, 12-1079-42, 1:400) (all from eBioscience); Brilliant Violet 510–anti-CD4 (RM4-5, 100559, 1:200), AF700–anti-CD8a (53-6.7, 100730, 1:200), Brilliant Violet 785–anti-TCRβ (H57-597, 109249, 1:200), PE–anti-CD45.2 (104, 109808, 1:400), PE/Dazzle 594–anti-PD-1 (29F.1A12, 135228, 1:400), Alexa Fluor 647–anti-granzyme B (GB11, 515405, 1:100), PE-Cyanine7–anti-IFNγ (XMG1.2, 505826, 1:200), Brilliant Violet 421–anti-TNFα (MP6-XT22, 506328, 1:200), APC–anti-IL-4 (11B11, 504106, 1:200), Pacific Blue–anti-IL-17A (TC11-18H10.1, 506918, 1:200), Brilliant Violet 711–anti-TIM-3 (RMT3-23, 119727, 1:400), Brilliant Violet 650–anti-CD44 (1M7, 103049, 1:400), PE-Cyanine7–anti-CD62L (MEL-14, 104417, 1:400), APC–anti-CD69 (H1.2F3, 104514, 1:200), Brilliant Violet 650–anti-CD11b (M1/70, 101259, 1:200), APC–anti-XCR1 (ZET, 148206, 1:400), Pacific Blue–anti-Ki67 (16A8, 652422, 1:400) (all from BioLegend); PE–anti-IL-2 (JES6-5H4, 554428, 1:200), Brilliant Violet 605–anti-Ly108 (13G3, 745250, 1:200) (from BD Biosciences); Alexa Fluor 647–anti-TCF1 (C63D9, 6709, 1:100, Cell Signaling Technology).

4. The following antibodies were used for immunoprecipitation or immunoblot analysis: anti-β-Actin (3700), anti-GAPDH (D16H11), anti-Lamin B1 (D4Q4Z), anti-HA (3724), anti-MIOS (13557), anti-WDR59 (53385) (all were used at 1:1,000 dilution and from Cell Signaling Technology); anti-Cathepsin D (AF1029, R&D); anti-FLCN (ab124885), anti-DEPDC5 (ab213181), anti-SEH1L (ab218531) (all were used at 1:1,000 and from Abcam); anti-SEC13 (sc-514308); anti-NPRL2 (sc-376986) (both were used at 1:1,000 and from Santa Cruz); anti-Flag (F1804, 1:1,000, Sigma); anti-TFEB (A303-673A, 1:1,000, Bethyl Laboratories); anti-SLC38A2 (BMP081, 1:1,000, MBL); anti-WDR24 (20778-1-AP, 1:1,000, ProteinTech); and anti-NPRL3 (NBP-97766, 1:1,000, Novus Biologicals). Primary antibodies were detected using HRP-conjugated anti-mouse IgG (W4021, Promega, 1:5,000).

5. The following antibodies or dyes for used for immunofluorescent imaging analysis: anti-EEA1 antibody (#3288, C45B10, 1:250; Cell Signaling Technology) followed by Alexa Fluor 488-conjugated anti-rabbit secondary antibody (A11008, 1:ug/ml; Thermo Fisher Scientific) or AlexaFluor 568-conjugated phalloidin to detect F-Actin (A12380, 1 U/ml; Thermo Fisher Scientific)

Validation

1. The following antibodies for flow cytometry have been validated for the specificity and application by the manufacturers (see detailed reference on the website)
7-AAD: https://www.sigmaaldrich.com/US/en/product/sigma/a9400
Fixable viability dye: https://www.thermofisher.com/order/catalog/product/65-0865-14?SID=srch-srp-65-0865-14
PE-Cy7–anti-CD11c: https://tonbobio.com/products/pe-cyanine7-anti-mouse-cd11c-n418
FITC–anti-FOXP3: https://www.thermofisher.com/antibody/product/FOXP3-Antibody-clone-FJK-16s-Monoclonal/11-5773-82
PE-Cyanine7–anti-T-bet: https://www.thermofisher.com/antibody/product/T-bet-Antibody-clone-eBio4B10-4B10-Monoclonal/25-5825-82
APC-eFluor 780–anti-MHC-II: https://www.thermofisher.com/antibody/product/MHC-Class-II-I-A-I-E-Antibody-clone-M5-114-15-2-Monoclonal/47-5321-82
PE-Cyanine7–anti-CD24: https://www.thermofisher.com/antibody/product/CD24-Antibody-clone-M1-69-Monoclonal/25-0242-82
FITC–anti-CD86: https://www.thermofisher.com/antibody/product/CD86-B7-2-Antibody-clone-GL1-Monoclonal/11-0862-82
PE–anti-IL-12/IL-23 p40: https://www.thermofisher.com/antibody/product/IL-12-IL-23-p40-Antibody-clone-C17-8-Monoclonal/12-7123-81
PE–anti-LAMP1: https://www.thermofisher.com/antibody/product/CD107a-LAMP-1-Antibody-clone-eBioH4A3-Monoclonal/12-1079-42
Brilliant Violet 510–anti-CD4: https://www.biolegend.com/en-us/products/brilliant-violet-510-anti-mouse-cd4-antibody-7991
AF700–anti-CD8a: https://www.biolegend.com/en-us/products/alexa-fluor-700-anti-mouse-cd8a-antibody-3387
Brilliant Violet 785–anti-TCRβ: https://www.biolegend.com/en-us/products/brilliant-violet-785-anti-mouse-tcr-b-chain-antibody-17614
PE–anti-CD45.2: https://www.biolegend.com/en-us/products/pe-anti-mouse-cd45-2-antibody-7
PE/Dazzle 594–anti-PD-1: https://www.biolegend.com/en-us/products/pe-dazzle-594-anti-mouse-cd279-pd-1-antibody-12090
Alexa Fluor 647–anti-granzyme B: https://www.biolegend.com/en-us/products/alexa-fluor-647-anti-human-mouse-granzyme-b-antibody-6067
PE-Cyanine7–anti-IFNγ: https://www.biolegend.com/en-us/products/pe-cyanine7-anti-mouse-ifn-gamma-antibody-5865
Brilliant Violet 421–anti-TNFα: https://www.biolegend.com/en-us/products/brilliant-violet-421-anti-mouse-tnf-alpha-antibody-7336

APC–anti-IL-4: https://www.biolegend.com/en-us/products/apc-anti-mouse-il-4-antibody-891
Pacific Blue–anti-IL-17A: https://www.biolegend.com/en-us/products/pacific-blue-anti-mouse-il-17a-antibody-4145
Brilliant Violet 711–anti-TIM-3: https://www.biolegend.com/en-us/products/brilliant-violet-711-anti-mouse-cd366-tim-3-antibody-14918
Brilliant Violet 650–anti-CD44: https://www.biolegend.com/en-us/products/brilliant-violet-650-anti-mouse-human-cd44-antibody-8923
PE-Cyanine7–anti-CD62L: https://www.biolegend.com/en-us/products/pe-cyanine7-anti-mouse-cd62l-antibody-1922
APC–anti-CD69: https://www.biolegend.com/en-us/products/apc-anti-mouse-cd69-antibody-3169
Brilliant Violet 650–anti-CD11b: https://www.biolegend.com/en-us/products/brilliant-violet-650-anti-mouse-human-cd11b-antibody-7638
APC–anti-XCR1: https://www.biolegend.com/en-us/products/apc-anti-mouse-rat-xcr1-antibody-10222
Pacific Blue–anti-Ki67: https://www.biolegend.com/en-us/products/pacific-blue-anti-mouse-ki-67-antibody-10553
PE–anti-IL-2: https://www.bdbiosciences.com/en-us/products/reagents/flow-cytometry-reagents/research-reagents/single-color-antibodies-ruo/pe-rat-anti-mouse-il-2.554428
Brilliant Violet 605–anti-Ly108: https://www.bdbiosciences.com/en-us/products/reagents/flow-cytometry-reagents/research-reagents/single-color-antibodies-ruo/bv605-mouse-anti-mouse-ly-108.745250
Alexa Fluor 647–anti-TCF1: https://www.cellsignal.com/products/antibody-conjugates/tcf1-tcf7-c63d9-rabbit-mab-alexa-fluor-647-conjugate/6709

2. The specificities of listed immunoblot antibodies have been validated by the manufacturer by western blot (see detailed information on the detailed reference on the website) or by in-house methods (see more details below):
anti-β-Actin (3700): https://www.cellsignal.com/products/primary-antibodies/b-actin-8h10d10-mouse-mab/3700
anti-GAPDH (D16H11): https://www.cellsignal.com/products/primary-antibodies/gapdh-d16h11-xp-rabbit-mab/5174
anti-Lamin B1 (D4Q4Z): https://www.cellsignal.com/products/primary-antibodies/lamin-b1-d4q4z-rabbit-mab/12586
anti-HA (3724): https://www.cellsignal.com/products/primary-antibodies/ha-tag-c29f4-rabbit-mab/3724
anti-MIOS (13557): https://www.cellsignal.com/products/primary-antibodies/mios-d12c6-rabbit-mab/13557
anti-WDR59 (53385): https://www.cellsignal.com/products/primary-antibodies/wdr59-d4z7a-rabbit-mab/53385
anti-EEA1 antibody (3288): https://www.cellsignal.com/products/primary-antibodies/eea1-c45b10-rabbit-mab/3288
anti-Cathepsin D (AF1029): https://www.rndsystems.com/products/mouse-cathepsin-d-antibody_af1029
anti-FLCN (ab124885): https://www.abcam.com/products/primary-antibodies/flcn-antibody-epncir147-ab124885.html
anti-DEPDC5 (ab213181): https://www.abcam.com/products/primary-antibodies/depdc5-antibody-epr20497-23-ab213181.html
anti-SEH1L (ab218531): https://www.abcam.com/products/primary-antibodies/seh1l-antibody-epr20851-ab218531.html
anti-SEC13 (sc-514308): https://www.scbt.com/p/sec13-antibody-f-6
anti-NPRL2 (sc-376986): https://www.scbt.com/p/nprl2-antibody-f-3
anti-Flag (F1804): https://www.sigmaaldrich.com/US/en/product/sigma/f1804
anti-TFEB (A303-673A): https://www.fortislife.com/products/primary-antibodies/rabbit-anti-tfeb-antibody/BETHYL-A303-673
anti-SLC38A2 (BMP081): https://www.mblbio.com/bio/g/dtl/A/?pcd=BMP081
anti-WDR24 (20778-1-AP): https://www.ptglab.com/products/WDR24-Antibody-20778-1-AP.htm
anti-NPRL3 (NBP-97766, Novus Biologicals): https://www.novusbio.com/products/nprl3-antibody_nbp1-97766

Among these antibodies, anti-SLC38A2 (BMP081, MBL) was also in-house validated by immunoblot analysis of Cas9-expressing MC38 or B16-OVA cell lines transduced with sgRNAs targeting SLC38A2 (or non-targeting control). Anti-FLCN (ab124885) and anti-TFEB (A303-673A, Bethyl Laboratories) were also in-house validated by immunoblot analysis of cDC1 from wild-type or Flcn/TfebΔDC mice.

Eukaryotic cell lines

Policy information about cell lines and Sex and Gender in Research

Cell line source(s)	HEK293T and B16F10 cell lines were purchased from ATCC. MC38, MC38-OVA and B16-OVA cell lines were kindly provided by Dr. Dario Vignali. B16-FLT3L cell line was kindly provided by Dr. Douglas R. Green. B16F10 cell line expressing ZsGreen (B16-ZsGreen) was generated in-house by lentiviral transduction of pHIV-ZsGreen construct (18121, Addgene) and were sorted for expression of ZsGreen.
Authentication	The cell lines used were not authenticated.
Mycoplasma contamination	The cell lines were not tested for mycoplasma contamination.
Commonly misidentified lines (See ICLAC register)	No commonly misidentified cell lines were used.

Animals and other research organisms

Policy information about studies involving animals; ARRIVE guidelines recommended for reporting animal research, and Sex and Gender in Research

Laboratory animals	Mice were housed and bred at the St. Jude Children's Research Hospital Animal Resource Center in specific pathogen-free conditions. Mice were on 12-hour light/dark cycles that coincide with daylight in Memphis, TN, USA. The St. Jude Children's Research Hospital Animal Resource Center housing facility was maintained at 20–25°C and 30–70 % humidity. C57BL/6, CD45.1+, OT-I, OT-II, Cas9-transgenic, Batf3−/−, Rag1−/−, Cd4Cre, CD11c-Cre and XCR1-Cre mice were purchased from The Jackson Laboratory. Slc38a2fl/fl mice were purchased from INFRAFRONTIER/EMMA. Flcnfl/fl mice were kindly provided by Laura Schmidt. Tfebfl/fl mice were kindly provided by Andrea Ballabio. The mice were backcrossed to the C57BL/6 background; sex- and age-matched mice were used throughout the study at 7–12 weeks old, and both male and female mice were used. The genetically modified mice were viable and developed normally. To generate mixed bone marrow chimaeras, bone marrow cells from WT or FlcnΔDC mice were mixed with cells

	from Batf3−/− mice at a 1:1 ratio and transferred into lethally irradiated (11 Gy) CD45.1+ mice. For chimeras used in Extended Data Fig. 5i–k and Fig. 8c–e, bone marrow cells from WT, Slc38a2ΔDC or FlcnΔDC mice were mixed with cells from CD45.1+ mice at a 1:1 ratio and transferred into lethally irradiated (11 Gy) C57BL/6 mice, followed by reconstitution for 6–8 weeks. In certain experiments, bone marrow cells from wild-type or FlcnΔDC mice were transferred into lethally irradiated (11 Gy) CD45.1+ mice.
Wild animals	The study did not involve wild animals.
Reporting on sex	Both male and female mice were included in all analyses reported in this manuscript, as there were no differences between sexes observed in any of our biological or functional assays.
Field-collected samples	The study did not involve samples collected from the field.
Ethics oversight	Experiments and procedures were performed in accordance with the Institutional Animal Care and Use Committee (IACUC) of St. Jude Children's Research Hospital.

Note that full information on the approval of the study protocol must also be provided in the manuscript.

Flow Cytometry

Plots

Confirm that:

☒ The axis labels state the marker and fluorochrome used (e.g. CD4-FITC).

☒ The axis scales are clearly visible. Include numbers along axes only for bottom left plot of group (a 'group' is an analysis of identical markers).

☒ All plots are contour plots with outliers or pseudocolor plots.

☒ A numerical value for number of cells or percentage (with statistics) is provided.

Methodology

Sample preparation	The spleens, peripheral lymph nodes (PLN) and mesenteric lymph nodes (MLN) were gently grinded under nylon mesh using the flat end of a 3-mL syringes. Red blood cells were removed by ACK lysing buffer, followed by washing cells with isolation buffer. For DC analysis and enrichment, mouse spleens were digested with 1 mg/ml collagenase IV plus 200 U/ml DNase I (DN25, Sigma) for 45 min at 37°C. To prepare intratumoral lymphocytes, tumors were harvested at day 15 or 19 after inoculation, excised, minced and digested with 1 mg/ml collagenase IV (Worthington) and 200 U/ml DNase I (Sigma) for 1 h at 37°C. For DC migration analysis, mouse tumor-draining lymph nodes (dLN) were digested with 1 mg/ml collagenase IV plus 200 U/ml DNase I (DN25, Sigma) for 30 min at 37°C.
Instrument	LSRII or LSR Fortessa (BD Biosciences); Reflection cell sorter (i-Cyt).
Software	BD FACSDIva software (version 8) was used to collect flow cytometry data on LSRII, Fortessa or Symphony A3 cytometers (BD Biosciences). FlowJo v10 (TreeStar) for FACS results.
Cell population abundance	The purities of the sorted cells were more than 98%.
Gating strategy	For all experiments, FSC-A vs. SSC-A gates was used to identify population targeted viable cells. Singlet cells were separated from doublets using FSC-A vs. FSC-H gating. Live viability dye was used to eliminate dead cells. Target populations were further determined by specific antibodies, which were able to distinguish from negative populations.

☒ Tick this box to confirm that a figure exemplifying the gating strategy is provided in the Supplementary Information.
